# Drinking Water Contaminant Exposures and Risk of Uterine Cancer

**DOI:** 10.1001/jamanetworkopen.2026.24391

**Published:** 2026-07-22

**Authors:** Maya Spaur, Lydia Marcus Post, Laura E. Beane Freeman, Danielle N. Medgyesi, Jared A. Fisher, Jessica M. Madrigal, Samantha Ammons, Emma S. Spielfogel, Komal Bangia, Alexander P. Keil, James V. Lacey, Mary H. Ward, Rena R. Jones

**Affiliations:** 1 Occupational and Environmental Epidemiology Branch, Division of Cancer Epidemiology and Genetics, National Cancer Institute, Rockville, Maryland; 2 Cancer Prevention Fellowship Program, Division of Cancer Prevention, National Cancer Institute, Rockville, Maryland; 3 Department of Environmental Health Sciences, Mailman School of Public Health, Columbia University, New York, New York; 4 Division of Health Analytics, Department of Computational and Quantitative Medicine, Beckman Research Institute, City of Hope, Duarte, California; 5 Community and Environmental Epidemiology Research Branch, Office of Environmental Health Hazard Assessment, Oakland, California

## Abstract

**Importance:**

Some drinking water contaminants, including disinfection byproducts, are known or suspected carcinogens with endocrine-disrupting properties. Few epidemiologic studies have evaluated associations with uterine cancer.

**Objective:**

To investigate prospective associations between multiple regulated contaminants in drinking water and the risk of uterine cancer.

**Design, Setting, and Participants:**

The California Teachers Study is a prospective cohort of female California educators. Participants who were cancer-free and had not undergone hysterectomy at enrollment (1995-1996) were followed up through December 31, 2020. Data were analyzed from November 2024 to October 2025.

**Exposures:**

The 15-year mean concentrations (1990-2005) of individual and total trihalomethanes (THMs) and haloacetic acids (HAAs), nitrate, arsenic, and uranium were calculated. The mixture effects of individual THMs, arsenic, uranium, and nitrate were also evaluated using quantile-based g-computation.

**Main Outcomes and Measures:**

Associations with incident uterine cancer overall, endometrioid tumors, and nonendometrioid tumors were analyzed categorically by tertile and continuously per log_2_ increase in water contaminant exposures, using Cox models adjusted for age, body mass index, and smoking status.

**Results:**

This study included 53 100 women (median [IQR] age, 50 [43-60] years) with enrollment addresses linked to a community water supply among women with residential duration at enrollment of 10 years or more. Higher levels of total THMs were associated with a higher risk of uterine cancer (hazard ratio [HR], 1.18 [95% CI, 1.02-1.38]; *P* for trend = .06) and endometrioid tumors specifically (HR, 1.23 [95% CI, 1.05-1.46]; *P* for trend = .01). Positive associations were found for the individual THM chloroform. Higher levels of the sum of 5 HAAs were associated with nonendometrioid tumors (HR_,_ 1.86 [95% CI, 1.06-3.25]; *P* for trend = .01), with similar associations for the HAA monochloroacetic acid. No associations were observed with nitrate, arsenic, or uranium levels. Associations per IQR increase in the drinking water contaminant mixture composed of individual THMs, nitrate, arsenic, and uranium were 1.21 (95% CI, 0.96-1.53) and 1.34 (95% CI, 1.04-1.74) for uterine and endometrioid histotypes, respectively. The THMs chloroform and dibromochloromethane were the major contributors.

**Conclusions and Relevance:**

In this cohort study of female California educators, associations between drinking water THMs and uterine cancer overall and with endometrioid tumors specifically were consistent with findings from the only prior study, conducted in the midwestern US. Further investigation of drinking water contaminants as modifiable risk factors for this common gynecologic cancer is warranted.

## Key Points

**Question:**

Is exposure to disinfection byproducts and inorganic contaminants in public water supplies associated with the risk of uterine cancer?

**Findings:**

In this cohort study of 53 100 female educators in California, exposure to higher levels of total trihalomethanes was associated with incident uterine cancer and endometrioid tumors specifically, with positive associations observed for some individual trihalomethanes. Higher levels of total haloacetic acids were associated with nonendometrioid tumors, while no associations were observed for nitrate, arsenic, or uranium.

**Meaning:**

These findings suggest that long-term exposure to certain disinfection byproducts may increase the risk of uterine cancer.

## Teaser

This cohort study examines the association between exposure to regulated drinking water contaminants and the risk of uterine cancer among female educators in California.

## Introduction

Uterine cancer is the most commonly diagnosed gynecologic cancer in the US, with an estimated 69 120 cases expected in 2025 (3.4% of all new cancers diagnosed); cancer of the endometrium accounts for more than 95% of all uterine cancer cases.^1^ Age-adjusted incidence and mortality rates for uterine cancer have increased in recent years in the US,^1^ and incidence rates have risen around the world since the 1990s.^2^ Disparities in advanced-stage endometrial cancer diagnoses and survival disproportionately impact Black women, who have a higher mortality rate than White women.^3,4^ Regional variation in diagnoses is also apparent. From 2010 to 2019, hysterectomy-corrected, state-specific incidence rates of endometrioid carcinoma, the predominant histologic type, were highest in the Midwestern US and Appalachia, and rates of nonendometrioid tumors were highest in the South, Northeast, and Midwest.^5^ The increasing trends and regional variation in rates suggest that environmental exposures may be risk factors.

Drinking water can be a major source of exposure to known or suspected carcinogenic contaminants, as well as those with endocrine-disrupting properties associated with reproductive toxicity.^6,7^ Approximately 90% of the US population gets its drinking water from community water systems (CWSs).^8^ The US Environmental Protection Agency regulates specific contaminants in CWSs by enforceable maximum contaminant levels that consider economic and technical feasibility and public health benefit for certain health outcomes.^9^ Commonly occurring regulated contaminants include inorganics (nitrate, arsenic, and uranium) and disinfection byproducts, including trihalomethanes (THMs) and haloacetic acids (HAAs).

A positive association was previously observed between disinfection byproducts in drinking water and uterine cancer in a single prior study, the Iowa Women’s Health Study (IWHS), a population-based prospective cohort of postmenopausal women; however, there was no association with nitrate concentrations.^10^ The International Agency for Research on Cancer classified specific disinfection byproducts as possibly carcinogenic to humans (Group 2B) based on insufficient evidence of carcinogenicity in epidemiologic studies and sufficient evidence in experimental animals: chloroform (bladder cancer in animals and humans), bromodichloromethane (liver and kidney tumorigenicity in animals; no epidemiologic evidence), and the HAAs dibromoacetic and trichloroacetic acids (hepatocarcinogenesis in animals).^11,12,13,14,15^ Our objective was to evaluate associations between uterine cancer incidence and CWS disinfection byproducts, nitrate, uranium, and arsenic exposures as single contaminants and as a mixture in the California Teachers Study (CTS), a cohort of female California teachers and administrators.

## Methods

### Study Population

The CTS was designed to investigate the etiology of breast and other cancers.^16^ In this cohort study, public school teachers and administrators in the California State Teachers Retirement System were mailed a self-administered questionnaire. A total of 133 476 participants completed the survey and enrolled between 1995 and 1996; 7 had baseline surveys with data discrepancies and were excluded.^16^ We further excluded participants who, at baseline, lived outside of California (n = 8332), only consented to breast cancer research (n = 18), or had a hysterectomy (n = 23 635) or a prevalent cancer (excluding nonmelanoma skin cancer) based on self-report of California Cancer Registry records (n = 13 603), resulting in 87 881 participants eligible for inclusion in this analysis (eFigure 1 in Supplement 1). Participants who did not develop uterine cancer were censored at the earliest of their date of move out of California, any other cancer diagnosis, a hysterectomy, death, or the end of follow-up (December 31, 2020). This cohort study was approved by the institutional review boards of the National Institutes of Health and the City of Hope, and completion of the questionnaires was considered to imply written informed consent. We describe our results based on the Strengthening the Reporting of Observational Studies in Epidemiology (STROBE) guidelines.

Participants self-reported information at enrollment (1995-1996) on sociodemographic characteristics (race and ethnicity, age), anthropometrics (height and weight, used to calculate body mass index [BMI] as weight in kilograms divided by height in meters squared), smoking and alcohol consumption, and personal medical history (menopause status, oral contraceptive use, total number of live births). Race and ethnicity were included to allow for an understanding of the generalizability of the results. For the 6% of women who did not report menopause status at baseline, we imputed this information as perimenopausal or postmenopausal if they were older than 52 years at enrollment, and as premenopausal if they were aged 52 years or younger. Hormone therapy use information was reported among perimenopausal and postmenopausal women (responses included no hormone therapy, past hormone therapy [all types], current estrogen only, current progesterone only, and current estrogen and progesterone); we dichotomized responses into ever or never hormone therapy use. Neighborhood characteristics of the enrollment addresses (quartiles of socioeconomic status and urbanicity dichotomized as metropolitan and nonmetropolitan areas) were previously developed using 1990 Census block group data.^17^

### Cancer and Mortality Ascertainment

Incident cancers were identified through linkage with the California Cancer Registry. Mortality information was obtained from linkage with the California state mortality file and Social Security Administration death master file. Uterine cancer was defined based on the *International Classification of Diseases for Oncology*, *Third Edition*, site code C541, and included histology codes that were grouped into histotypes: endometrioid, nonendometrioid, and other.

### Drinking Water Data and Exposure Assessment

We obtained a geospatial dataset of statewide drinking water boundaries, as well as CWS monitoring data (1990-2020) for THMs, HAAs, arsenic, uranium, and nitrate-nitrogen (nitrate-N), from the California Office of Environmental Health Hazard Assessment.^18,19,20,21^ THMs included the individual species chloroform, dibromochloromethane, bromodichloromethane, and bromoform, which are regulated as total THMs (TTHM); and HAAs included monochloroacetic acid, dichloroacetic acid, trichloroacetic acid, monobromoacetic acid, and dibromoacetic acid, regulated as the sum of 5 HAAs (HAA5).^7^ Mean annual concentrations of each contaminant were computed for each CWS. Measurements from samples of treated or delivered drinking water were prioritized; when treated samples were not available, we averaged results from raw or untreated samples for the inorganic contaminants.^18^ Specific procedures for individual contaminants and handling of nondetects are published^22^ and described in the eMethods in Supplement 1.

Residential histories were previously constructed for CTS participants.^23^ At enrollment, geocoded addresses for 81 093 participants (92%) were linked to 1127 CWS distribution boundaries.^18,21,23,24,25^ The 6788 participants whose enrollment address did not link to a CWS were assumed to be domestic well users and were excluded from this study.

For our main analyses, we excluded 25 166 participants on a CWS who resided less than 10 years at the enrollment address, based on self-reported information about residential duration from the fourth questionnaire and the constructed residential history.^23^ Excluded women were similar to those included, although excluded participants tended to be younger and a greater proportion were nulliparous (eTable 1 in Supplement 1). We computed 15-year mean contaminant concentrations (1990-2005) for CWSs linked to the enrollment address, excluding 2827 participants missing TTHM measurements for a total of 53 100 in our main analyses, constituting a total of 1 032 131 person-years, with a mean follow-up of 19.4 years and 913 CWSs. Most participants (>93%) had 1990-2005 CWS arsenic (n = 53 060), nitrate (n = 53 073), and uranium (n = 49 831) data. We had HAA data for a small number of CWSs before 2013, as locational annual mean concentration monitoring was in effect for all systems by October 1, 2013, per the Stage 2 Disinfection Byproducts Rule (eFigure 2 in Supplement 1).^26^ We calculated mean concentrations for HAAs from 1990 to 2013; for HAA analyses, we started follow-up on January 1, 2010, and excluded participants who were censored before that date (n = 13 200). For comparability, we calculated mean concentrations for THMs from 1990 to 2013, with follow-up starting in 2010.

Alternatively, we calculated yearly mean estimates of CWS contaminants for all residence-years that linked to a CWS starting at enrollment and including residential changes during follow-up (n = 76 821). Participants with any residence located outside of a CWS service area were assumed to be on a domestic well and excluded.

### Statistical Analysis

Data were analyzed from November 2024 to October 2025. We described participant characteristics and contaminant exposures overall and among all uterine cancer cases (n = 1038) and the endometrioid (n = 864) and nonendometrioid (n = 154) histotypes. We evaluated Spearman rank correlation coefficients (ρ) between contaminant concentrations.

We used Cox proportional hazards regression models, with time-on-study as the time scale, to estimate hazard ratios (HRs) and 95% CIs between drinking water contaminant exposures and all uterine carcinomas and endometrioid and nonendometrioid cases separately, with 2-sided tests of statistical significance (Type I error α = .05 without correction for multiple comparisons). We did not reject the proportional hazards assumption based on graphical analysis of Schoenfeld residuals. We parameterized mean drinking water exposures in 2 ways: continuously after log_2_ transformation and in tertiles. In categorical analyses, we tested for trend using the median of each tertile parametrized as a continuous variable. We evaluated potential nonlinearity in associations using cubic splines. Models were adjusted for potential confounders and other known risk factors derived from a directed acyclic graph (eFigure 3 in Supplement 1): baseline age and baseline age squared, BMI category (<25, 25-<30, ≥30, or missing), and smoking status (never, former, current, or missing). In supplemental analyses, we additionally adjusted for reproductive factors: menopausal status (premenopausal, perimenopausal or postmenopausal, or missing), ever had live births (no, yes, or missing), and oral contraceptive use (never, ever, or missing).

To evaluate the joint effects of the drinking water contaminant mixture, we used quantile-based g-computation via the qgcomp package in R.^27,28^ Estimates were log_2_ transformed and divided by the IQR to standardize concentrations. We estimated HRs per IQR increase in the contaminant mixture. We explored mixtures composed of different combinations of contaminants that were adjusted for the contaminants not included in the mixture. Separately, we evaluated the single effect of each contaminant, adjusted for all others.

In addition to the mean metric from 1990 to 2005, we estimated associations for time-varying cumulative mean concentrations lagged 5 years for participants whose complete residential history (100% of residence-years) was linked to a CWS. For comparison to the main results, we conducted a sensitivity analysis further restricted to participants included in the main analytic population (ie, lived at the enrollment address for at least 10 years). In ad hoc analyses, we evaluated cumulative mean concentrations lagged 10 years, starting follow-up on January 1, 2000, and excluding participants censored before that date. We adjusted for age as a time-varying covariate in these models; other covariates were identical to the main analyses.

Because the 15-year mean concentrations at the enrollment address in our main analyses overlapped with the first 10 years of follow-up, we performed a sensitivity analysis in which we started follow-up on January 1, 2005, and excluded participants who were censored before that date. Separately, to confirm whether the associations were consistent when mean exposures were below current regulatory limits, we evaluated single contaminant models and mixture analyses among participants whose 15-year mean exposure levels were below the maximum contaminant levels (n = 49 394). To account for potential clustering in exposures by CWS, we evaluated a Cox mixed effects model with a random effect for the CWS identifier.

We stratified our main analyses by smoking, BMI, and menopausal status to explore potential interactions between water contaminants and these factors. We tested statistical interaction using the likelihood ratio test to determine statistical heterogeneity for all stratified analyses, deriving a *P* value from the χ^2^ test statistic. Smoking is inversely associated with uterine cancer risk,^29^ while a higher BMI, hormone therapy use, and being postmenopausal are risk factors.^30^ Among perimenopausal and postmenopausal participants at baseline (n = 25 050), we further stratified by never vs ever use of hormone therapy. All statistical analyses were conducted in R, version 4.3.3 (R Project for Statistical Computing) within the CTS Researcher Platform.^31^

## Results

The study included 53 100 participants (median [IQR] age, 50 [43-60] years). Among participants, 1% identified as American Indian or Alaska Native, 4% as Asian or Pacific Islander, 3% as Black, 5% as Hispanic, 85% as White, and 1% as other race or multiracial. Approximately half (48%) of participants were premenopausal at baseline (Table 1). More than 50% of participants had BMI less than 25, were never smokers, consumed less than 20 g of alcohol per day, and lived in metropolitan areas or census block groups above the 50th percentile of socioeconomic status. Participants who developed uterine cancer were less likely to report ever using oral contraceptives (57%) than the full cohort (68%).

**Table 1.** Descriptive Statistics of California Teachers Study (CTS) Participants^a^

Characteristic	All (N = 53 100)	Uterine cancer (n = 1038)	Endometrioid (n = 864)	Nonendometrioid (n = 154)
Age, median (IQR), y	50 (43-60)	54 (48-64)	54 (48-64)	54 (48-63)
Menopause status, No. (%)				
Premenopausal	25 495 (48)	375 (36)	309 (36)	58 (38)
Perimenopausal or postmenopausal	24 156 (45)	599 (58)	501 (58)	90 (58)
Missing	3449 (6)	64 (6)	54 (6)	6 (4)
Live births, No. (%)				
0	13 035 (25)	290 (28)	255 (30)	30 (19)
≥1	38 899 (73)	724 (70)	588 (68)	121 (79)
Missing	1166 (2)	24 (2)	21 (2)	3 (2)
BMI category, No. (%)				
<25	31 430 (59)	460 (44)	388 (45)	65 (42)
25 to <30	12 515 (24)	272 (26)	222 (26)	46 (30)
≥30	7168 (13)	270 (26)	222 (26)	39 (25)
Missing	1987 (4)	36 (3)	32 (4)	4 (3)
Oral contraceptive use, No. (%)				
Never	15 331 (29)	406 (39)	345 (40)	56 (36)
Ever	35 944 (68)	594 (57)	489 (57)	90 (58)
Missing	1825 (3)	38 (4)	30 (3)	8 (5)
Hormone therapy use, No. (%)				
Never	7483 (30)	158 (26)	120 (24)	35 (41)
Ever	17 567 (33)	442 (43)	383 (44)	50 (32)
Missing	28 050 (53)	438 (42)	361 (42)	69 (45)
Race and ethnicity, No. (%)^b^				
American Indian or Alaska Native	365 (1)	5 (<1)	3 (<1)	1 (1)
Asian or Pacific Islander	2354 (4)	51 (5)	40 (5)	11 (7)
Black	1431 (3)	23 (2)	14 (2)	8 (5)
Hispanic	2509 (5)	33 (3)	29 (3)	4 (3)
White	45 333 (85)	909 (88)	764 (88)	128 (83)
Other or multiracial	647 (1)	8 (1)	6 (1)	1 (1)
Not reported	461 (1)	9 (1)	8 (1)	1 (1)
Smoking status, No. (%)				
Never	35 484 (67)	693 (67)	570 (66)	112 (73)
Former	14 659 (28)	303 (29)	262 (30)	36 (23)
Current	2645 (5)	39 (4)	29 (3)	6 (4)
Missing	312 (1)	3 (<1)	3 (<1)	0
Alcohol consumption, No. (%), g/d				
0	16 946 (34)	374 (37)	302 (36)	61 (40)
<20	29 490 (58)	543 (54)	456 (55)	80 (53)
≥20	4128 (8)	85 (8)	73 (9)	10 (7)
SES quartile, No. (%)				
First (low)	1792 (3)	34 (3)	30 (4)	4 (3)
Second	7877 (15)	152 (15)	124 (15)	25 (16)
Third	17 433 (33)	368 (36)	311 (36)	50 (32)
Fourth (high)	25 693 (49)	475 (46)	390 (46)	75 (49)
Urbanicity, No. (%)				
Nonmetropolitan	16 666 (32)	344 (33)	289 (34)	44 (29)
Metropolitan	36 141 (68)	685 (67)	566 (66)	110 (71)
Mean CWS concentration, median (IQR)^c^				
TTHM, μg/L	4.56 (0.32-20.7)	5.16 (0.56-20.84)	5.16 (0.56-21.49)	4.79 (0.39-17.15)
Chloroform, μg/L	2.79 (0.31-7.67)	3.17 (0.46-9.08)	3.25 (0.47-9.08)	2.81 (0.24-6.86)
Bromoform, μg/L	0.16 (0.08-1.49)	0.16 (0.08-1.49)	0.18 (0.08-1.61)	0.15 (0.08-1.49)
Bromodichloromethane, μg/L	1.08 (0.16-6.29)	1.41 (0.23-6.29)	1.45 (0.27-6.29)	1.04 (0.15-6.02)
Dibromochloromethane, μg/L	0.67 (0.12-3.7)	0.82 (0.13-3.71)	0.84 (0.14-4.28)	0.72 (0.09-2.9)
Arsenic, μg/L	0.99 (0.59-1.8)	0.97 (0.59-1.8)	0.97 (0.59-1.8)	0.99 (0.59-1.77)
Uranium, μg/L	3.79 (1.35-6.39)	3.68 (1.03-6.29)	3.64 (1.04-6.29)	4.09 (1.11-6.32)
Nitrate-N, mg/L	0.57 (0.22-2.24)	0.53 (0.22-2.12)	0.52 (0.22-2.15)	0.58 (0.19-2.11)
HAA5, μg/L	7.97 (3.01-15.23)	7.51 (3.28-15.37)	7.32 (3.48-15.23)	7.82 (3.74-15.87)

Abbreviations: BMI, body mass index (calculated as weight in kilograms divided by height in meters squared); CWS, community water system; HAA5, total haloacetic acids; N, nitrogen; SES, socioeconomic status; THMs, trihalomethanes; TTHM, total trihalomethanes.

^a^ CWS exposures were linked to address at enrollment. All characteristics are based on self-report at enrollment. Analyses were restricted to participants with a residential duration at enrollment of 10 years or more, and TTHM estimates.

^b^ Participant race and ethnicity were self-reported at enrollment, and categorized by the CTS as follows: American Indian or Alaska Native (American Indian or Alaska Native only or White and American Indian or Alaska Native reported), Asian or Pacific Islander (Asian or Pacific Islander only [Chinese, Filipino, Hawaiian, Japanese, Vietnamese, or Korean] or White and Asian or Pacific Islander reported), Black (Black only or White and Black reported), Hispanic (Hispanic only or White and Hispanic reported), White (only non-Hispanic White reported), or other race or multiracial (other reported, or more than 1 of the aforementioned groups reported).

^c^ CWS exposures represent 15-year mean concentrations (1990-2005) for arsenic (n = 53 060), uranium (n = 49 831), nitrate-N (n = 53 073), individual THMs and TTHM, and a mean concentration for HAA5 from 1990 to 2013.

We observed modest differences and overlapping distributions in TTHM concentrations among all participants and those who developed uterine cancer (Table 1). Nitrate-N, arsenic, and uranium concentrations were moderately positively correlated in pairwise analyses (Spearman ρ = 0.32-0.47) and negatively correlated with concentrations of individual THMs and TTHM, though uranium was positively correlated with bromoform (ρ = 0.22) (eFigure 4 in Supplement 1). Individual THMs were positively correlated with each other and TTHM (ρ = 0.31-0.91). Similarly, individual HAAs were positively correlated with each other and HAA5 (ρ = 0.18-0.94).

In multivariable analyses, a doubling in TTHM concentrations was associated with uterine cancer overall (HR, 1.02 [95% CI, 1.00-1.04]) (Figure). For categorical exposures, the highest risk was evident among women at and above the third tertile (16 μg/L) compared with the lowest tertile (≤1.2 μg/L) (HR, 1.18 [95% CI, 1.02-1.38]; *P* for trend = .06) (Table 2). We observed positive associations between TTHM and risk of endometrioid tumors (HR, 1.23 [95% CI, 1.05-1.46]; *P* for trend = .01), but no associations with nonendometrioid tumors (HR, 0.95 [95% CI, 0.63-1.42]; *P* for trend = .50). Among individual THMs, we observed the highest HRs for uterine cancer overall and endometrioid tumors with chloroform (HR, 1.18 [95% CI, 1.02-1.37]; *P* for trend = .07 and HR, 1.27 [95% CI, 1.07-1.50]; *P* for trend = .01, respectively). No associations were observed with 15-year mean arsenic, nitrate-N, or uranium exposures (Table 3). Additional adjustment for baseline reproductive factors (menopausal status, oral contraceptive use, live births) did not appreciably change risk estimates (eTable 2 in Supplement 1). Exposure-response associations using cubic splines were consistent with those in the categorical analyses (eFigures 5 and 6 in Supplement 1).

**Figure.**
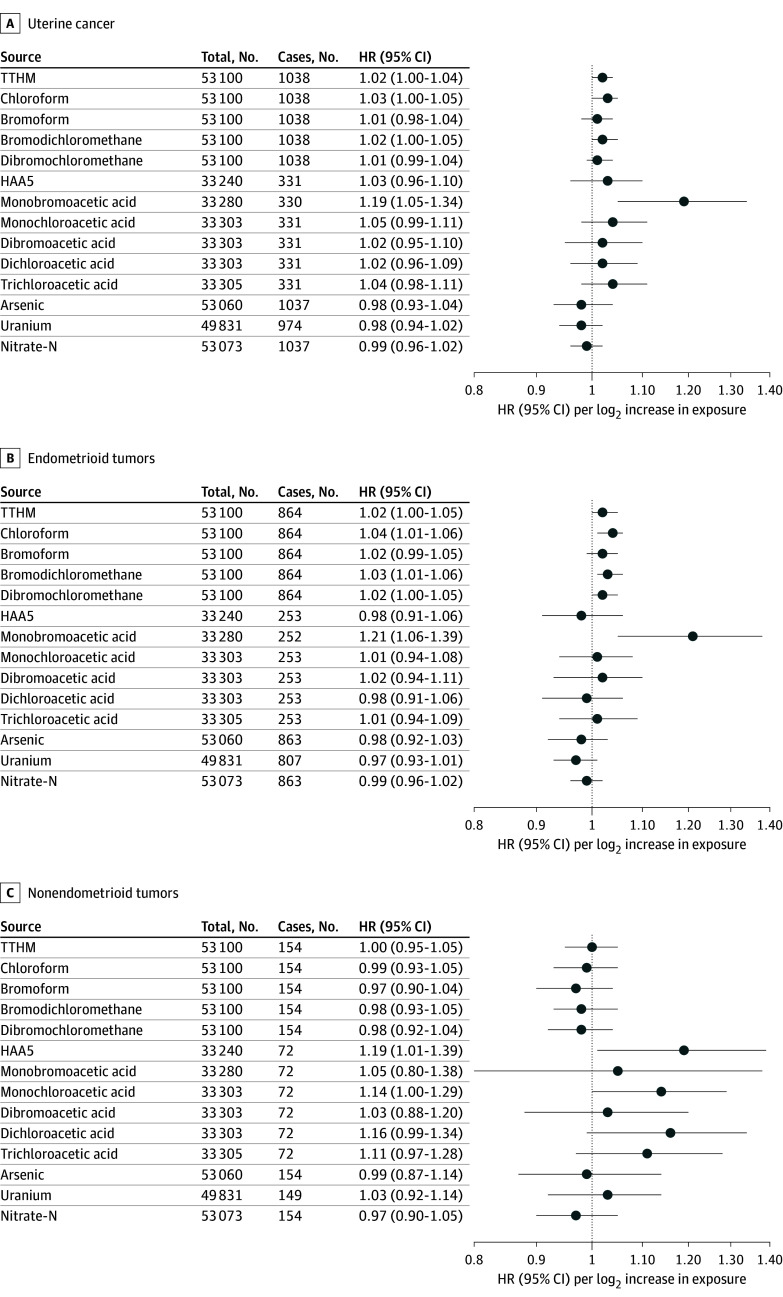
Forest Plots of Hazard Ratios (HRs) for Uterine Cancer, Endometrioid Tumors, and Nonendometrioid Tumors Per Log_2_ Increase in Exposures The figure shows the hazard ratios of uterine cancer (A), endometrioid tumors (B), and nonendometrioid tumors (C) per log_2_ increase in community water system exposures. Community water system exposures were linked to the address at enrollment and represent mean concentrations of trihalomethanes and inorganics from 1990 to 2005, and mean concentrations of haloacetic acids from 1990 to 2013. Haloacetic acid analyses started follow-up in 2010. Analyses were restricted to participants with a residential duration at enrollment of 10 years or more. The model was adjusted for baseline age (years) and baseline age squared, body mass index category (calculated as weight in kilograms divided by height in meters squared [<25, 25-<30, ≥30, or missing]) at baseline, and smoking status at baseline (never, former, current, or missing). Dots represent the HR, and horizontal lines represent the 95% CIs. A dashed vertical line represents where HR = 1. HAA5 indicates total haloacetic acids; nitrate-N, nitrate-nitrogen; TTHM, total trihalomethanes.

**Table 2.** Hazard Ratios (HRs) of Uterine Cancer, Endometrioid Tumors, and Nonendometrioid Tumors Per Tertile of Trihalomethane Exposures^a^^,^^b^

Exposure per tertile of CWS concentration	Total, No.	Uterine cancer		Endometrioid		Nonendometrioid	
		Cases, No.	HR (95% CI)^c^	Cases, No.	HR (95% CI)^c^	Cases, No.	HR (95% CI)^c^
**TTHM, μg/L**							
T1 (0-1.22)	17 571	307	1 [Reference]	253	1 [Reference]	48	1 [Reference]
T2 (1.23-15.57)	17 825	355	1.11 (0.96-1.30)	287	1.09 (0.92-1.29)	59	1.19 (0.81-1.74)
T3 (15.58-108.66)	17 704	376	1.18 (1.02-1.38)	324	1.23 (1.05-1.46)	47	0.95 (0.63-1.42)
*P* for trend^d^	NA	NA	.06	NA	.01	NA	.50
**Chloroform, μg/L**							
T1 (0-0.65)	17 570	303	1 [Reference]	245	1 [Reference]	52	1 [Reference]
T2 (0.66-5.77)	17 728	360	1.14 (0.98-1.33)	294	1.15 (0.97-1.37)	57	1.06 (0.73-1.54)
T3 (5.78-82.55)	17 802	375	1.18 (1.02-1.38)	325	1.27 (1.07-1.50)	45	0.83 (0.56-1.24)
*P* value trend^d^	NA	NA	.07	NA	.01	NA	.25
**Bromoform, μg/L**							
T1 (0-0.10)	17 586	350	1 [Reference]	285	1 [Reference]	57	1 [Reference]
T2 (0.11-0.62)	17 620	331	0.96 (0.82-1.11)	276	0.98 (0.83-1.16)	47	0.83 (0.57-1.22)
T3 (0.63-8.78)	17 894	357	1.00 (0.87-1.16)	303	1.05 (0.89-1.23)	50	0.86 (0.59-1.26)
*P* for trend^d^	NA	NA	.71	NA	.45	NA	.70
**Bromodichloromethane, μg/L**							
T1 (0-0.35)	17 695	314	1 [Reference]	250	1 [Reference]	54	1 [Reference]
T2 (0.36-3.23)	17 592	357	1.15 (0.99-1.34)	301	1.22 (1.03-1.44)	51	0.97 (0.66-1.42)
T3 (3.24-20.92)	17 813	367	1.14 (0.98-1.32)	313	1.22 (1.03-1.44)	49	0.89 (0.60-1.31)
*P* for trend^d^	NA	NA	.27	NA	.11	NA	.54
**Dibromochloromethane, μg/L**							
T1 (0-0.20)	17 638	323	1 [Reference]	256	1 [Reference]	57	1 [Reference]
T2 (0.21-1.69)	17 661	357	1.09 (0.94-1.27)	304	1.17 (0.99-1.38)	48	0.84 (0.57-1.23)
T3 (1.70-17.85)	17 801	358	1.08 (0.93-1.26)	304	1.16 (0.98-1.37)	49	0.84 (0.58-1.24)
*P* for trend^d^	NA	NA	.54	NA	.30	NA	.58

Abbreviations: BMI, body mass index (calculated as weight in kilograms divided by height in meters squared); CWS, community water system; NA, not applicable; T, tertile; THMs, trihalomethanes; TTHM, total trihalomethanes.

^a^ CWS exposures were linked to the address at enrollment and represent mean concentrations of individual THMs and TTHM from 1990 to 2005.

^b^ Analyses were restricted to participants with a residential duration at enrollment of 10 years or more.

^c^ Model was adjusted for baseline age (years) and baseline age squared, BMI category (<25, 25-<30, >30, or missing), and smoking status (never, former, current, or missing).

^d^ *P* for trend was evaluated using the median of each tertile.

**Table 3.** Hazard Ratios (HRs) of Uterine Cancer, Endometrioid Tumors, and Nonendometrioid Tumors Per Tertiles of Arsenic, Uranium, and Nitrate-Nitrogen (N) Exposures^a^^,^^b^

Exposure per tertile of CWS concentration	Total, No.	Uterine cancer		Endometrioid		Nonendometrioid	
		Cases, No.	HR (95% CI)^c^	Cases, No.	HR (95% CI)^c^	Cases, No.	HR (95% CI)^c^
**Arsenic, μg/L**							
T1 (0.01-0.70)	17 507	365	1 [Reference]	304	1 [Reference]	54	1 [Reference]
T2 (0.71-1.61)	16 760	306	0.90 (0.77-1.05)	256	0.91 (0.77-1.07)	47	0.93 (0.63-1.38)
T3 (1.62-36.55)	18 793	366	0.95 (0.83-1.10)	303	0.95 (0.81-1.11)	53	0.93 (0.64-1.36)
*P* for trend^d^	NA	NA	.75	NA	.71	NA	.75
**Uranium, μg/L**							
T1 (0.09-2.63)	16 592	343	1 [Reference]	288	1 [Reference]	47	1 [Reference]
T2 (2.64-5.18)	16 600	317	0.98 (0.84-1.14)	264	0.97 (0.82-1.15)	49	1.10 (0.74-1.65)
T3 (5.19-100.93)	16 639	314	0.94 (0.80-1.09)	255	0.91 (0.77-1.07)	53	1.15 (0.78-1.70)
*P* for trend^d^	NA	NA	.39	NA	.24	NA	.52
**Nitrate-N, mg/L**							
T1 (0.01-0.25)	17 539	351	1 [Reference]	289	1 [Reference]	57	1 [Reference]
T2 (0.26-1.53)	17 814	352	0.98 (0.85-1.14)	299	1.02 (0.86-1.19)	45	0.77 (0.52-1.13)
T3 (1.54-9.62)	17 720	334	0.95 (0.81-1.10)	275	0.95 (0.80-1.12)	52	0.90 (0.61-1.31)
*P* value trend^d^	NA	NA	.46	NA	.43	NA	.91

Abbreviations: BMI, body mass index (calculated as weight in kilograms divided by height in meters squared); CWS, community water system; NA, not applicable; T, tertile.

^a^ CWS exposures were linked to the address at enrollment and represent mean concentrations of arsenic, uranium, and nitrate-N from 1990 to 2005.

^b^ Analyses were restricted to participants with a residential duration at enrollment of 10 years or more.

^c^ Model was adjusted for baseline age (years) and baseline age squared, BMI category (<25, 25-<30, >30, or missing), and smoking status (never, former, current, or missing).

^d^ *P* for trend was evaluated using the median of each tertile.

In analyses of individual and HAA5 exposures (1990-2013 mean), a doubling in monobromoacetic acid levels was associated with elevated risk of uterine cancer (HR, 1.19 [95% CI, 1.05-1.34]) and endometrioid tumors (HR, 1.21 [95% CI, 1.06-1.39]) (Figure). An increased risk of nonendometrioid tumors was observed at higher levels of HAA5 (HR, 1.86 [95% CI, 1.06-3.25]; *P* for trend = .01), and monochloroacetic acid was also positively associated with risk (HR, 1.86 [95% CI, 1.01-3.44]; *P* for trend = .05) (Table 4).

**Table 4.** Hazard Ratios (HRs) of Uterine Cancer, Endometrioid Tumors, and Nonendometrioid Tumors Per Tertiles of Haloacetic Acid Exposures^a^^,^^b^

Exposure per tertile of CWS concentration	Total, No.	Uterine cancer		Endometrioid		Nonendometrioid	
		Cases, No.	HR (95% CI)^c^	Cases, No.	HR (95% CI)^c^	Cases, No.	HR (95% CI)^c^
**HAA5, μg/L**							
T1 (0.35-4.60)	11 098	104	1 [Reference]	84	1 [Reference]	19	1 [Reference]
T2 (4.61-13.10)	10 965	106	1.07 (0.81-1.40)	87	1.08 (0.8-1.46)	18	1.00 (0.52-1.91)
T3 (13.11-115.18)	11 177	121	1.18 (0.91-1.53)	82	0.99 (0.73-1.35)	35	1.86 (1.06-3.25)
*P* for trend^d^	NA	NA	.21	NA	.89	NA	.01
**Monobromoacetic acid, μg/L**							
T1 (0.02-0.25)	11 119	107	1 [Reference]	79	1 [Reference]	27	1 [Reference]
T2 (0.26-0.30)	10 571	98	0.98 (0.75-1.29)	76	1.03 (0.75-1.41)	21	0.84 (0.48-1.49)
T3 (0.31-4.60)	11 590	125	1.13 (0.88-1.47)	97	1.19 (0.88-1.60)	24	0.87 (0.50-1.51)
*P* for trend^d^	NA	NA	.29	NA	.23	NA	.67
**Monochloroacetic acid, μg/L**							
T1 (0-0.10)	9931	101	1 [Reference]	85	1 [Reference]	15	1 [Reference]
T2 (0.11-0.26)	11 940	108	0.88 (0.67-1.15)	82	0.79 (0.59-1.07)	25	1.38 (0.73-2.62)
T3 (0.27-6.78)	11 432	122	1.05 (0.81-1.37)	86	0.88 (0.65-1.19)	32	1.86 (1.01-3.44)
*P* for trend^d^	NA	NA	.36	NA	.80	NA	.05
**Dibromoacetic acid, μg/L**							
T1 (0.03-0.73)	11 054	106	1 [Reference]	80	1 [Reference]	24	1 [Reference]
T2 (0.74-2.55)	10 468	105	1.04 (0.79-1.36)	78	1.02 (0.75-1.39)	26	1.16 (0.66-2.02)
T3 (2.56-30.0)	11 781	120	1.07 (0.83-1.39)	95	1.12 (0.83-1.51)	22	0.88 (0.49-1.57)
*P* for trend^d^	NA	NA	.60	NA	.42	NA	.57
**Dichloroacetic acid, μg/L**							
T1 (0.03-1.82)	11 016	105	1 [Reference]	85	1 [Reference]	18	1 [Reference]
T2 (1.83-5.60)	11 030	115	1.11 (0.86-1.45)	91	1.09 (0.81-1.46)	23	1.31 (0.71-2.43)
T3 (5.61-38.41)	11 257	111	1.06 (0.81-1.38)	77	0.91 (0.67-1.24)	31	1.71 (0.96-3.06)
*P* for trend^d^	NA	NA	.76	NA	.47	NA	.07
**Trichloroacetic acid, μg/L**							
T1 (0.04-1.32)	11 113	102	1 [Reference]	83	1 [Reference]	18	1 [Reference]
T2 (1.33-4.34)	11 013	118	1.19 (0.91-1.55)	90	1.11 (0.83-1.50)	27	1.54 (0.85-2.8)
T3 (4.35-87.78)	11 179	111	1.11 (0.84-1.45)	80	0.98 (0.72-1.34)	27	1.51 (0.83-2.74)
*P* for trend^d^	NA	NA	.58	NA	.81	NA	.25

Abbreviations: BMI, body mass index (calculated as weight in kilograms divided by height in meters squared); CWS, community water system; HAA5, total haloacetic acids; NA, not applicable.

^a^ CWS exposures were linked to the address at enrollment and represent mean concentrations of individual and HAA5 from 1990 to 2013.

^b^ Analyses were restricted to participants with a residential duration at enrollment of 10 years or more. Participants who were censored before 2010 were excluded. Person-time was recalculated to determine follow-up time started on January 1, 2010, and age was recalculated to reflect age on January 1, 2010 (baseline age plus years between the participant start date and January 1, 2010). The total person-years was 308 457, with a mean follow-up of 9.3 years.

^c^ Model was adjusted for age in 2010 (years) and baseline age squared, BMI category (<25, 25-<30, >30, or missing), and smoking status (never, former, current, or missing).

^d^ *P* for trend was evaluated using the median of each tertile.

### Mixture Models

In analyses of joint associations, HRs per IQR increase in the contaminant mixture of individual THMs, arsenic, uranium, and nitrate were 1.21 (95% CI, 0.96-1.53) for uterine cancer overall and 1.34 (95% CI, 1.04-1.74) for endometrioid tumors (eTable 3 in Supplement 1). Chloroform and dibromochloromethane were the largest contributors to the positive mixture association with uterine cancer and the endometrioid type; bromoform and bromodichloromethane were the largest contributors to the positive association with nonendometrioid tumors. Associations were slightly attenuated in the mixture containing TTHM (eTable 3 in Supplement 1). In single contaminant analyses mutually adjusted for others, TTHM had the highest HRs for uterine cancer (HR, 1.13 [95% CI, 0.99-1.29]) and endometrioid tumors specifically (HR, 1.18 [95% CI, 1.02-1.37]), and uranium was associated with nonendometrioid tumors (HR, 1.13 [95% CI, 0.84-1.51]). In mixture analyses including HAA5, we observed positive associations for uterine cancer overall and for nonendometrioid tumors (eTable 4 in Supplement 1).

### Stratified Analyses

Findings were similar across BMI categories for most contaminants, although the highest HRs for uterine cancer overall and endometrioid tumors were observed among participants with a BMI less than 25 per doubling in chloroform levels (eTable 5 in Supplement 1). We did not observe clear differences in associations or statistical interaction (*P* for interaction >.05) by menopausal status (eTable 6 in Supplement 1), smoking status (eTable 7 in Supplement 1) or hormone therapy use (eTable 8 in Supplement 1).

### Sensitivity Analyses

Patterns of association with time-varying cumulative mean exposures lagged 5 and 10 years were generally consistent with our main analysis, although effect estimates were slightly attenuated (eTables 9-11 in Supplement 1). In analyses with follow-up starting on January 1, 2005, we observed similar patterns and higher HRs than in our main analyses (eTable 12 in Supplement 1). Modeling THMs as mean concentrations from 1990 to 2013 (similar to the HAA exposure metrics) also generated similar findings, although associations with nonendometrioid tumors were positive at higher levels of TTHM and for chloroform (eTable 13 in Supplement 1). In analyses restricted to participants whose 15-year mean contaminant exposures were below their respective maximum contaminant levels, the findings were also consistent (eTable 14 in Supplement 1). Results from Cox mixed effects models were robust compared with our main analyses using Cox proportional hazards models (eTable 15 in Supplement 1).

## Discussion

In a large prospective cohort of female educators in California, we observed positive associations between the common public drinking water contaminants TTHM, chloroform, and bromodichloromethane and uterine cancer, and with endometrioid tumors specifically. Our mixture analysis was also showed a positive association. Although we had more limited exposure data for HAAs, we observed positive associations with monobromoacetic acid and uterine cancer overall and endometrioid tumors, whereas HAA5 and monochloroacetic acid were positively associated with nonendometrioid tumors. We did not observe associations with arsenic, uranium, or nitrate. To our knowledge, this is the first epidemiologic evaluation of the association of both single and co-occurring exposures to nitrate, uranium, arsenic, and disinfection byproducts with the development of this cancer.

Few epidemiologic studies have evaluated associations between drinking water contaminants and uterine cancer risk, and only 1 prior study investigated disinfection byproducts.^10^ In the IWHS cohort of postmenopausal women across Iowa, relatively high levels of exposure to both TTHM and HAA5 in public drinking water (≥93.0 μg/L vs <0.9 μg/L) were associated with increased endometrial cancer risk (HR [95% CI], TTHM: 2.19 [1.41-3.40]; HAA5: 1.84 [1.19-2.83]; *P* for trend <.01).^10^ Although long-term mean exposure levels were somewhat lower in the CTS (median [95th percentile], TTHM: 4.6 [38.3] μg/L; HAA5: 8.0 [30.0] μg/L) than IWHS (median [95th percentile], TTHM: 4.8 [93.2] μg/L; HAA5: 1.9 [48.5] μg/L), we found associations generally consistent with those in the IWHS. Our mixture analyses signaled TTHM as a potential driver of the associations with uterine cancer overall and for endometrioid tumors, although the marginal statistical significance and limited precision of these analyses should be considered.

The observed associations with disinfection byproducts are biologically plausible. In experimental studies, certain THMs and HAAs have been shown to bind to estrogen receptors and have endocrine-disrupting properties that can cause hormonal dysregulation, a hypothesized mechanism for uterine cancer development.^32,33^ Cellular evidence has shown that brominated THMs (but not chloroform) can cause mutagenicity under activation by glutathione *S-*transferase theta 1.^34,35^ Chloroform is likely to be carcinogenic to humans via cytotoxicity rather than genotoxicity.^36^ Monobromoacetic and monochloroacetic acids have been found to cause oxidative stress and genotoxicity in human cells,^37^ whereas dichloroacetic acid and trichloroacetic acid reduced estrogen receptor levels in mice.^38^ Our stratified analyses were motivated by established relationships of smoking and uterine cancer (generally inverse) and higher adiposity and hormone therapy use (positive). However, we did not observe differences in the observed associations by smoking status, similar to findings in the IWHS.^10^ We also found no differences by hormone therapy use, whereas the IWHS reported an interaction between ever use and TTHM on endometrial cancer risk.^10^ We observed modest differences in endometrioid tumor associations across BMI strata for chloroform but not for any other contaminants.

### Strengths and Limitations

Strengths of our study include the prospective study design with more than 20 years of follow-up of a geographically diverse population; information on key confounders, hysterectomy, and uterine cancer histology; and water quality data for common contaminants. The water quality data had high agreement with self-reported questionnaire information in this cohort.^21^ We also evaluated uterine cancer associations among premenopausal and postmenopausal women separately, potentially important because hormone-related and reproductive risk factors differ by menopausal status.^39^

This study also has some limitations. Our analyses lacked water quality data before 1990, which limited our ability to assess early life exposures or longer latency periods that may be relevant to uterine cancer risk.^40^ We partly addressed this limitation by restricting our analyses to a residentially stable subpopulation with 10 years or more at their enrollment address. Further, regulatory changes for some contaminants (eg, arsenic, disinfection byproducts) were implemented after 2005, indicating that mean concentrations from 1990 to 2005 may be reflective of pre-1990 exposures. The patterns of association in time-varying analyses were generally consistent with those incorporating long-term mean exposures, although effect estimates were attenuated. This finding could possibly be explained by measurement error in our time-independent exposures compared with the time-varying measures or indicate critical windows of exposure for these relationships that are not well captured with long-term mean concentrations. Additional limitations include our exposure assessment based on residential address rather than self-reported drinking water source; however, we previously demonstrated high agreement between self-reported and assigned water source among a subset at follow-up.^21^ We also did not have information on water consumption; however, disinfection byproduct measures from the participant’s CWS may nonetheless reflect exposures among those who did not drink their home tap water but were exposed via inhalation and dermal routes, especially during showering or bathing.^34^

The CTS includes primarily teachers. Almost all participants had at least a college education, and few lived in Census block groups with socioeconomic status indexes below the 25th quartile. Both our study population and California’s teachers have higher percentages of individuals from minoritized racial and ethnic groups than California’s population overall. Further, the generalizability of our findings to other populations may be limited. Although we evaluated major confounders from the literature, residual confounding may still exist.

We excluded women from the main analysis for several reasons, including shorter residential duration at their enrollment address and addresses that did not link to a CWS. Private wells are not regulated nor routinely monitored for contaminants, and therefore we could not explore equivalent measures of exposure among the 8% of participants who likely used private wells. However, characteristics between participants included in and those excluded from our analyses showed them to be largely similar.

## Conclusions

In this cohort study of female California educators, we observed novel associations between THM exposures in drinking water and the risk of uterine cancer, particularly endometrioid histotypes. We also observed associations between HAAs and nonendometrioid tumors, although these findings should be interpreted cautiously considering the limited number of historical estimates of these exposures. Uterine cancer is the sixth most diagnosed malignancy in women worldwide, and increasing incidence trends motivate research into exogenous risk factors. Exposure to drinking water contaminants is potentially modifiable, underscoring the need for future research and attention to this important public health issue.

## Notes

Supplement 1. **eMethods**. **eTable 1.** Characteristics of California Teachers Study Participants, Comparing Participants Excluded and Included in the Main Analytic Study Population **eTable 2.** Hazard Ratios Per Doubling and Tertiles of Exposure, Additionally Adjusted for Reproductive Factors **eTable 3.** Hazard Ratios Per Increase in the Exposure Mixture, and Per Single Contaminants Mutually Adjusted **eTable 4.** Hazard Ratios Per Increase in the Exposure Mixture Including Haloacetic Acids **eTable 5.** Hazard Ratios Per Doubling in Exposures, Stratified by Body Mass Index Category **eTable 6.** Hazard Ratios Per Doubling in Exposures, Stratified by Menopausal Status **eTable 7.** Hazard Ratios Per Doubling in Exposures, Stratified by Smoking Status **eTable 8.** Hazard Ratios Per Doubling in Exposures, Stratified by Hormone Therapy Use **eTable 9.** Hazard Ratios Per Doubling and Tertiles of Time-Varying Exposures, Lagged 5 Years **eTable 10.** Hazard Ratios Per Doubling and Tertiles of Time-Varying Exposures, Lagged 5 Years Among Main Analytic Population **eTable 11.** Hazard Ratios Per Doubling and Tertiles of Time-Varying Exposures, Lagged 10 Years **eTable 12.** Hazard Ratios Per Doubling and Tertiles of Exposures, Excluding Participants Censored Prior to 2005 **eTable 13.** Hazard Ratios Per Doubling and Tertiles of 1990-2013 Average Trihalomethanes Exposures **eTable 14.** Hazard Ratios Per Doubling in Exposures Among Participants With Exposures Less Than Maximum Contaminant Levels **eTable 15.** Hazard Ratios Per Doubling and Tertiles of Exposures Using Mixed-Effects Models **eFigure 1.** Exclusion and Inclusion Criteria **eFigure 2.** Number of Community Water System Reporting Annual Water Quality Data by Year and Contaminant **eFigure 3.** Directed Acyclic Graph of Exposures, Outcomes, and Confounders **eFigure 4.** Spearman Correlation Coefficients of Community Water System Concentrations **eFigure 5.** Exposure-Response Associations Between Community Water System Nitrate, Uranium, Arsenic, Total and Individual Trihalomethanes and Uterine Cancer **eFigure 6.** Exposure-Response Associations Between Community Water System Nitrate, Uranium, Arsenic, Total and Individual Trihalomethanes and Endometrioid Tumors
eReferences

Supplement 2.
Data Sharing Statement
