# Supplementary material for: Drinking Water Contaminant Exposures and Risk of Uterine Cancer
Source: JAMA Netw Open. 2026 Jul 22;9(7):e2624391. doi: 10.1001/jamanetworkopen.2026.24391 (PMC13392805; doi:10.1001/jamanetworkopen.2026.24391)
Supplement: Supplement 1. — eMethods. eTable 1. Characteristics of California Teachers Study Participants, Comparing Participants Excluded and Included in the Main Analytic Study Population eTable 2. Hazard Ratios Per Doubling and Tertiles of Exposure, Additionally Adjusted for Reproductive Factors eTable 3. Hazard Ratios Per Increase in the Exposure Mixture, and Per Single Contaminants Mutually Adjusted eTable 4. Hazard Ratios Per Increase in the Exposure Mixture Including Haloacetic Acids eTable 5. Hazard Ratios Per Doubling in Exposures, Stratified by Body Mass Index Category eTable 6. Hazard Ratios Per Doubling in Exposures, Stratified by Menopausal Status eTable 7. Hazard Ratios Per Doubling in Exposures, Stratified by Smoking Status eTable 8. Hazard Ratios Per Doubling in Exposures, Stratified by Hormone Therapy Use eTable 9. Hazard Ratios Per Doubling and Tertiles of Time-Varying Exposures, Lagged 5 Years eTable 10. Hazard Ratios Per Doubling and Tertiles of Time-Varying Exposures, Lagged 5 Years Among Main Analytic Population eTable 11. Hazard Ratios Per Doubling and Tertiles of Time-Varying Exposures, Lagged 10 Years eTable 12. Hazard Ratios Per Doubling and Tertiles of Exposures, Excluding Participants Censored Prior to 2005 eTable 13. Hazard Ratios Per Doubling and Tertiles of 1990-2013 Average Trihalomethanes Exposures eTable 14. Hazard Ratios Per Doubling in Exposures Among Participants With Exposures Less Than Maximum Contaminant Levels eTable 15. Hazard Ratios Per Doubling and Tertiles of Exposures Using Mixed-Effects Models eFigure 1. Exclusion and Inclusion Criteria eFigure 2. Number of Community Water System Reporting Annual Water Quality Data by Year and Contaminant eFigure 3. Directed Acyclic Graph of Exposures, Outcomes, and Confounders eFigure 4. Spearman Correlation Coefficients of Community Water System Concentrations eFigure 5. Exposure-Response Associations Between Community Water System Nitrate, Uranium, Arsenic, Total and Individual Trihalomethanes and Uterine Cance [file jamanetwopen-e2624391-s001.pdf]

# Supplemental Online Content

Spaur M, Marcus Post L, Beane Freeman LE, et al. Drinking water contaminant exposures and risk of uterine cancer in the California Teachers Study cohort. *JAMA Netw Open*. 2026;9(7):e2624391. doi:10.1001/jamanetworkopen.2026.24391

**eMethods.**
**eTable 1.** Characteristics of California Teachers Study Participants, Comparing Participants Excluded and Included in the Main Analytic Study Population
**eTable 2.** Hazard Ratios Per Doubling and Tertiles of Exposure, Additionally Adjusted for Reproductive Factors
**eTable 3.** Hazard Ratios Per Increase in the Exposure Mixture, and Per Single Contaminants Mutually Adjusted
**eTable 4.** Hazard Ratios Per Increase in the Exposure Mixture Including Haloacetic Acids
**eTable 5.** Hazard Ratios Per Doubling in Exposures, Stratified by Body Mass Index Category
**eTable 6.** Hazard Ratios Per Doubling in Exposures, Stratified by Menopausal Status
**eTable 7.** Hazard Ratios Per Doubling in Exposures, Stratified by Smoking Status
**eTable 8.** Hazard Ratios Per Doubling in Exposures, Stratified by Hormone Therapy Use
**eTable 9.** Hazard Ratios Per Doubling and Tertiles of Time-Varying Exposures, Lagged 5 Years
**eTable 10.** Hazard Ratios Per Doubling and Tertiles of Time-Varying Exposures, Lagged 5 Years Among Main Analytic Population
**eTable 11.** Hazard Ratios Per Doubling and Tertiles of Time-Varying Exposures, Lagged 10 Years
**eTable 12.** Hazard Ratios Per Doubling and Tertiles of Exposures, Excluding Participants Censored Prior to 2005
**eTable 13.** Hazard Ratios Per Doubling and Tertiles of 1990-2013 Average Trihalomethanes Exposures
**eTable 14.** Hazard Ratios Per Doubling in Exposures Among Participants With Exposures Less Than Maximum Contaminant Levels
**eTable 15.** Hazard Ratios Per Doubling and Tertiles of Exposures Using Mixed-Effects Models
**eFigure 1.** Exclusion and Inclusion Criteria
**eFigure 2.** Number of Community Water System Reporting Annual Water Quality Data by Year and Contaminant
**eFigure 3.** Directed Acyclic Graph of Exposures, Outcomes, and Confounders
**eFigure 4.** Spearman Correlation Coefficients of Community Water System Concentrations
**eFigure 5.** Exposure-Response Associations Between Community Water System Nitrate, Uranium, Arsenic, Total and Individual Trihalomethanes and Uterine Cancer
**eFigure 6.** Exposure-Response Associations Between Community Water System Nitrate, Uranium, Arsenic, Total and Individual Trihalomethanes and Endometrioid Tumors
**eReferences**

© 2026 Spaur M et al. JAMA Network Open.

This supplemental material has been provided by the authors to give readers additional information about their work.

© 2026 Spaur M et al. JAMA Network Open.

**eMethods**

Drinking water data and exposure assessment

For disinfection byproducts (DBPs), measurements were from post-treatment sample points collected within the distribution systems. Uranium concentrations were converted from pCi/L to µg/L using 1.49 as the conversion factor (pCi/L*1.49=µg/L). Community water systems (CWS) are required to report non-detections and concentrations when above the detection limits for the purposes of reporting (DLR). Samples marked as "below the DLR", or with concentrations of zero, or values unlikely to reflect true concentrations (i.e., equal to DLR or ½ DLR), were assigned a value based on a single imputation that used Tobit regression, existing measurement data, and assumed a log-normal distribution.[1] The upper bound for imputation was derived from the median of reported concentrations below the DLR.[1,2]

In addition to the CWS exposures linked by the enrollment address, we also linked participants' addresses from their post-enrollment residential history over the follow-up period to their corresponding CWS(s). Years with missing data were interpolated using monitoring data within the three compliance cycles, as previously described.[2]

© 2026 Spaur M et al. JAMA Network Open.

**eTable 1. Characteristics of California Teachers Study participants, comparing participants excluded and included in the main analytic study population.**

| | Excluded | Included |
|---|---|---|
| **N** | 34781 | 53100 |
| **Incident uterine cases, N (%)** | 502 (1) | 1038 (2) |
| **Incident endometrioid cases, N (%)** | 410 (1) | 864 (2) |
| **Incident non endometrioid cases, N (%)** | 78 (<0.1) | 154 (<0.1) |
| **Age, years, Median (IQR)** | 45 (35,56) | 50 (43,60) |
| **Menopause status, N (%)** | | |
| Pre | 21077 (61) | 25495 (48) |
| Peri/Post | 12060 (35) | 24156 (45) |
| Missing | 1644 (5) | 3449 (6) |
| **N Live Births, N (%)** | | |
| 0 | 12040 (35) | 13035 (25) |
| 1+ | 21949 (63) | 38899 (73) |
| Missing | 792 (2) | 1166 (2) |
| **BMI category, N (%)** | | |
| <25 kg/m$^2$ | 21965 (63) | 31430 (59) |
| 25-<30 kg/m$^2$ | 7362 (21) | 12515 (24) |
| >=30 kg/m$^2$ | 4106 (12) | 7168 (13) |
| Missing | 1348 (4) | 1987 (4) |
| **Oral contraceptive use, N (%)** | | |
| Never | 9252 (27) | 15331 (29) |
| Ever | 24177 (70) | 35944 (68) |
| Missing | 1352 (4) | 1825 (3) |
| **Hormone Therapy Use, N (%)** | | |
| Never | 3866 (32) | 7483 (30) |
| Ever | 8360 (24) | 17567 (33) |
| Missing | 26241 (76) | 28050 (53) |
| **Race and ethnicity[a], N (%)** | | |
| Asian | 1113 (3) | 2354 (4) |
| Black | 665 (2) | 1431 (3) |
| Hispanic | 1798 (5) | 2509 (5) |
| Native American | 318 (1) | 365 (1) |
| Not reported | 292 (1) | 461 (1) |
| White | 30004 (86) | 45333 (85) |
| Other/multi-racial | 591 (2) | 647 (1) |
| **Smoking status, N (%)** | | |
| Never | 24096 (69) | 35484 (67) |
| Former | 8762 (25) | 14659 (28) |
| Current | 1700 (5) | 2645 (5) |
| Missing | 223 (1) | 312 (1) |
| **Alcohol consumption, N (%)** | | |
| 0 g/d | 10875 (33) | 16946 (34) |
| <20 g/d | 19186 (59) | 29490 (58) |

© 2026 Spaur M et al. JAMA Network Open.

| | | |
|---|---|---|
| >=20 g/d | 2484 (8) | 4128 (8) |
| **SES Quartile, N (%)** | | |
| SES 1 (Low) | 1985 (6) | 1792 (3) |
| SES 2 | 7140 (21) | 7877 (15) |
| SES 3 | 11297 (34) | 17433 (33) |
| SES 4 (High) | 13275 (39) | 25693 (49) |
| **Urbanicity, N (%)** | | |
| Not metropolitan | 13878 (41) | 16666 (32) |
| Metropolitan | 19844 (59) | 36141 (68) |
| **Water source, N (%)** | | |
| CWS | 27993 (80) | 53100 (100) |
| Private well | 6788 (20) | 0 (0) |

[a]Participant race and ethnicity were self-reported at enrollment, and categorized by the CTS as follows: Asian/Pacific Islander (Asian/Pacific Islander only [Chinese, Filipino, Hawaiian, Japanese, Vietnamese, or Korean] or white and Asian/Pacific Islander), Black (Black only or white and Black reported), Hispanic (Hispanic only or white and Hispanic reported), Native American (Native American only or white and Native American reported), non-Hispanic white (only white reported), or Other/multi-racial (Other reported or more than one of the aforementioned groups reported).

© 2026 Spaur M et al. JAMA Network Open.

**eTable 2. Hazard Ratios (HR, 95% CIs) of uterine, endometrioid and non-endometrioid cancers per log2 concentration and tertiles of drinking water contaminant exposures[a,b] , additionally adjusted for reproductive factors.**

| CWS Concentration Range | N | Uterine Cancer N Cases | Uterine Cancer HR (95% CI)[c] | Endometrioid Tumors N Cases | Endometrioid Tumors HR (95% CI)[c] | Non-Endometrioid Tumors N Cases | Non-Endometrioid Tumors HR (95% CI)[c] |
|---|---|---|---|---|---|---|---|
| **TTHM** (μg/L) | | | | | | | |
| Log2 | 53100 | 1038 | 1.02 (1.00, 1.04) | 864 | 1.02 (1.00, 1.05) | 154 | 1.00 (0.95, 1.05) |
| T1 (0,1.22) | 17571 | 307 | 1.0 (reference) | 253 | 1.0 (reference) | 48 | 1.0 (reference) |
| T2 (1.25,15.57) | 17825 | 355 | 1.11 (0.95, 1.29) | 287 | 1.09 (0.92, 1.29) | 59 | 1.20 (0.82, 1.76) |
| T3 (15.83,108.66) | 17704 | 376 | 1.18 (1.02, 1.38) | 324 | 1.23 (1.05, 1.46) | 47 | 0.95 (0.64, 1.42) |
| P trend[d] | | | 0.05 | | 0.01 | | 0.50 |
| **Chloroform** (μg/L) | | | | | | | |
| Log2 | 53100 | 1038 | 1.03 (1.01, 1.05) | 864 | 1.04 (1.01, 1.06) | 154 | 0.99 (0.94, 1.05) |
| T1 (0,0.65) | 17570 | 303 | 1.0 (reference) | 245 | 1.0 (reference) | 52 | 1.0 (reference) |
| T2 (0.65,5.77) | 17728 | 360 | 1.14 (0.98, 1.33) | 294 | 1.15 (0.97, 1.36) | 57 | 1.07 (0.73, 1.55) |
| T3 (5.81,82.55) | 17802 | 375 | 1.19 (1.02, 1.38) | 325 | 1.27 (1.08, 1.50) | 45 | 0.83 (0.56, 1.24) |
| P trend[d] | | | 0.06 | | 0.009 | | 0.26 |
| **Bromoform** (μg/L) | | | | | | | |
| Log2 | 53100 | 1038 | 1.01 (0.98, 1.04) | 864 | 1.02 (0.99, 1.05) | 154 | 0.97 (0.90, 1.04) |
| T1 (0,0.10) | 17586 | 350 | 1.0 (reference) | 285 | 1.0 (reference) | 57 | 1.0 (reference) |
| T2 (0.10,0.62) | 17620 | 331 | 0.95 (0.82, 1.11) | 276 | 0.98 (0.83, 1.15) | 47 | 0.83 (0.56, 1.22) |
| T3 (0.62,8.78) | 17894 | 357 | 1.00 (0.86, 1.16) | 303 | 1.04 (0.88, 1.22) | 50 | 0.86 (0.59, 1.26) |
| P trend[d] | | | 0.75 | | 0.49 | | 0.72 |
| **Bromodichloromethane** (μg/L) | | | | | | | |
| Log2 | 53100 | 1038 | 1.02 (1.00, 1.05) | 864 | 1.03 (1.01, 1.06) | 154 | 0.98 (0.93, 1.05) |
| T1 (0,0.35) | 17695 | 314 | 1.0 (reference) | 250 | 1.0 (reference) | 54 | 1.0 (reference) |
| T2 (0.36,3.23) | 17592 | 357 | 1.15 (0.99, 1.34) | 301 | 1.22 (1.03, 1.44) | 51 | 0.97 (0.66, 1.42) |
| T3 (3.29,20.92) | 17813 | 367 | 1.14 (0.98, 1.32) | 313 | 1.21 (1.03, 1.43) | 49 | 0.89 (0.60, 1.31) |
| P trend[d] | | | 0.28 | | 0.11 | | 0.55 |
| **Dibromochloromethane** (μg/L) | | | | | | | |
| Log2 | 53100 | 1038 | 1.01 (0.99, 1.04) | 864 | 1.02 (1.00, 1.05) | 154 | 0.98 (0.92, 1.04) |
| T1 (0,0.20) | 17638 | 323 | 1.0 (reference) | 256 | 1.0 (reference) | 57 | 1.0 (reference) |
| T2 (0.20,1.69) | 17661 | 357 | 1.09 (0.93, 1.26) | 304 | 1.16 (0.99, 1.37) | 48 | 0.84 (0.57, 1.23) |
| T3 (1.70,17.85) | 17801 | 358 | 1.08 (0.93, 1.25) | 304 | 1.15 (0.97, 1.36) | 49 | 0.85 (0.58, 1.24) |
| P trend[d] | | | 0.59 | | 0.34 | | 0.59 |
| **Arsenic** (μg/L) | | | | | | | |
| Log2 | 53060 | 1037 | 0.98 (0.93, 1.04) | 863 | 0.97 (0.92, 1.03) | 154 | 0.99 (0.87, 1.13) |
| T1 (0.01,0.70) | 17507 | 365 | 1.0 (reference) | 304 | 1.0 (reference) | 54 | 1.0 (reference) |
| T2 (0.70,1.61) | 16760 | 306 | 0.90 (0.77, 1.05) | 256 | 0.90 (0.76, 1.07) | 47 | 0.93 (0.63, 1.37) |
| T3 (1.62,36.55) | 18793 | 366 | 0.95 (0.82, 1.10) | 303 | 0.95 (0.81, 1.11) | 53 | 0.92 (0.63, 1.35) |
| P trend[d] | | | 0.74 | | 0.71 | | 0.72 |
| **Uranium** (μg/L) | | | | | | | |
| Log2 | 49831 | 974 | 0.98 (0.94, 1.02) | 807 | 0.97 (0.92, 1.01) | 149 | 1.02 (0.92, 1.14) |
| T1 (0.09,2.63) | 16592 | 343 | 1.0 (reference) | 288 | 1.0 (reference) | 47 | 1.0 (reference) |
| T2 (2.64,5.18) | 16600 | 317 | 0.97 (0.83, 1.13) | 264 | 0.96 (0.82, 1.14) | 49 | 1.10 (0.73, 1.64) |
| T3 (5.20,100.93) | 16639 | 314 | 0.93 (0.80, 1.08) | 255 | 0.90 (0.76, 1.06) | 53 | 1.14 (0.77, 1.69) |
| P trend[d] | | | 0.34 | | 0.21 | | 0.54 |
| **Nitrate-N** (mg/L) | | | | | | | |

© 2026 Spaur M et al. JAMA Network Open.

| | | | | | | | |
|---|---|---|---|---|---|---|---|
| Log2 | 53073 | 1037 | 0.99 (0.96, 1.02) | 863 | 0.99 (0.96, 1.02) | 154 | 0.97 (0.89, 1.04) |
| T1 (0.01,0.25) | 17539 | 351 | 1.0 (reference) | 289 | 1.0 (reference) | 57 | 1.0 (reference) |
| T2 (0.25,1.53) | 17814 | 352 | 0.99 (0.85, 1.14) | 299 | 1.02 (0.87, 1.20) | 45 | 0.76 (0.51, 1.13) |
| T3 (1.53,9.62) | 17720 | 334 | 0.95 (0.81, 1.10) | 275 | 0.95 (0.81, 1.12) | 52 | 0.89 (0.61, 1.29) |
| P trend[d] | | | 0.47 | | 0.44 | | 0.88 |
| **HAA5** (μg/L) | | | | | | | |
| Log2 | 33240 | 331 | 1.03 (0.96, 1.10) | 253 | 0.98 (0.91, 1.06) | 72 | 1.19 (1.01, 1.39) |
| T1 (0.35,4.60) | 11098 | 104 | 1.0 (reference) | 84 | 1.0 (reference) | 19 | 1.0 (reference) |
| T2 (4.64,13.10) | 10965 | 106 | 1.06 (0.81, 1.39) | 87 | 1.07 (0.79, 1.44) | 18 | 1.00 (0.53, 1.91) |
| T3 (13.20,115.18) | 11177 | 121 | 1.18 (0.91, 1.54) | 82 | 0.99 (0.73, 1.35) | 35 | 1.86 (1.06, 3.26) |
| P trend[d] | | | 0.20 | | 0.90 | | 0.01 |
| **Monobromoacetic acid** (μg/L) | | | | | | | |
| Log2 | 33280 | 330 | 1.19 (1.05, 1.34) | 252 | 1.21 (1.06, 1.39) | 72 | 1.05 (0.80, 1.38) |
| T1 (0.02,0.25) | 11119 | 107 | 1.0 (reference) | 79 | 1.0 (reference) | 27 | 1.0 (reference) |
| T2 (0.25,0.30) | 10571 | 98 | 0.98 (0.74, 1.29) | 76 | 1.02 (0.75, 1.40) | 21 | 0.85 (0.48, 1.50) |
| T3 (0.30,4.60) | 11590 | 125 | 1.14 (0.88, 1.48) | 97 | 1.19 (0.89, 1.61) | 24 | 0.88 (0.51, 1.52) |
| P trend[d] | | | 0.26 | | 0.21 | | 0.69 |
| **Monochloroacetic acid** (μg/L) | | | | | | | |
| Log2 | 33303 | 331 | 1.05 (0.99, 1.11) | 253 | 1.01 (0.94, 1.08) | 72 | 1.14 (1.00, 1.29) |
| T1 (0,0.10) | 9931 | 101 | 1.0 (reference) | 85 | 1.0 (reference) | 15 | 1.0 (reference) |
| T2 (0.10,0.26) | 11940 | 108 | 0.88 (0.67, 1.16) | 82 | 0.80 (0.59, 1.08) | 25 | 1.37 (0.72, 2.60) |
| T3 (0.27,6.78) | 11432 | 122 | 1.06 (0.81, 1.38) | 86 | 0.89 (0.66, 1.20) | 32 | 1.86 (1.00, 3.43) |
| P trend[d] | | | 0.34 | | 0.85 | | 0.05 |
| **Dibromoacetic acid** (μg/L) | | | | | | | |
| Log2 | 33303 | 331 | 1.02 (0.95, 1.10) | 253 | 1.02 (0.94, 1.11) | 72 | 1.03 (0.88, 1.20) |
| T1 (0.03,0.73) | 11054 | 106 | 1.0 (reference) | 80 | 1.0 (reference) | 24 | 1.0 (reference) |
| T2 (0.75,2.55) | 10468 | 105 | 1.05 (0.80, 1.37) | 78 | 1.03 (0.75, 1.41) | 26 | 1.15 (0.66, 2.00) |
| T3 (2.56,30.0) | 11781 | 120 | 1.08 (0.83, 1.40) | 95 | 1.13 (0.84, 1.52) | 22 | 0.88 (0.49, 1.57) |
| P trend[d] | | | 0.58 | | 0.40 | | 0.58 |
| **Dichloroacetic acid** (μg/L) | | | | | | | |
| Log2 | 33303 | 331 | 1.02 (0.96, 1.09) | 253 | 0.98 (0.91, 1.06) | 72 | 1.16 (0.99, 1.34) |
| T1 (0.03,1.82) | 11016 | 105 | 1.0 (reference) | 85 | 1.0 (reference) | 18 | 1.0 (reference) |
| T2 (1.84,5.60) | 11030 | 115 | 1.11 (0.85, 1.44) | 91 | 1.08 (0.80, 1.45) | 23 | 1.31 (0.70, 2.42) |
| T3 (5.71,38.41) | 11257 | 111 | 1.06 (0.81, 1.38) | 77 | 0.91 (0.67, 1.24) | 31 | 1.70 (0.95, 3.05) |
| P trend[d] | | | 0.76 | | 0.48 | | 0.07 |
| **Trichloroacetic acid** (μg/L) | | | | | | | |
| Log2 | 33305 | 331 | 1.04 (0.98, 1.11) | 253 | 1.01 (0.94, 1.09) | 72 | 1.11 (0.97, 1.28) |
| T1 (0.04,1.32) | 11113 | 102 | 1.0 (reference) | 83 | 1.0 (reference) | 18 | 1.0 (reference) |
| T2 (1.35,4.34) | 11013 | 118 | 1.18 (0.91, 1.54) | 90 | 1.11 (0.82, 1.49) | 27 | 1.54 (0.85, 2.8) |
| T3 (4.37,87.78) | 11179 | 111 | 1.11 (0.84, 1.45) | 80 | 0.98 (0.72, 1.34) | 27 | 1.50 (0.82, 2.72) |
| P trend[d] | | | 0.58 | | 0.82 | | 0.26 |

[a]Community water system (CWS) exposures were linked to the address at enrollment and represent 1990-2005 average concentrations of individual and total trihalomethanes (TTHM) and inorganics, and 1990-2013 average concentrations of individual and total haloacetic acids (HAA5).

[b]Analyses were restricted to participants with a residential duration at enrollment ≥ 10 years. For haloacetic acid analyses, participants who were censored prior to 2010 were excluded and person time was re-calculated so that follow-up time started on Jan 1, 2010, and age was re-calculated to reflect age on Jan 1, 2010.

© 2026 Spaur M et al. JAMA Network Open.

[c]Model was adjusted for baseline age (years) and age$^2$, BMI category (<25 kg/m$^2$, 25-<30 kg/m$^2$, >30 kg/m$^2$, or missing), smoking status (never, former, current, or missing), menopause status (pre- or peri/post-menopause), oral contraceptive use (never/ever/missing), and live births (no/yes/missing).
[d]P trend was evaluated using the median of each tertile.

© 2026 Spaur M et al. JAMA Network Open.

**eTable 3. Hazard ratios (HR, 95% CI) for uterine, endometrioid, and non-endometroid cancers per interquartile range (IQR) increase in the exposure mixture[a], and per single contaminants mutually adjusted[b].**

| | Uterine Cancer N=974 | Endometrioid Tumors N=807 | Non-Endometrioid Tumors N=149 |
|---|---|---|---|
| | **HR (95% CI)[c]** | **HR (95% CI)[c]** | **HR (95% CI)[c]** |
| **Joint effect of mixture, N=49,831** | | | |
| Individual THMs & Inorganics Mixture (Chloroform + Bromoform + Bromodichloromethane + Dibromochloromethane + Arsenic + Uranium + Nitrate-N) | 1.21 (0.96, 1.53) | 1.34 (1.04, 1.74) | 0.67 (0.36, 1.24) |
| TTHM & Inorganics Mixture (TTHM + Arsenic + Uranium + Nitrate-N) | 1.14 (0.91, 1.41) | 1.19 (0.93, 1.51) | 0.86 (0.50, 1.50) |
| Individual THMs Mixture (Chloroform + Bromoform + Bromodichloromethane + Dibromochloromethane) | 1.12 (0.98, 1.29) | 1.20 (1.03, 1.39) | 0.88 (0.61, 1.26) |
| Brominated THMs Mixture (Bromoform + Bromodichloromethane + Dibromochloromethane) + Adjust for Chloroform | 1.13 (0.97, 1.32) | 1.21 (1.02, 1.44) | 0.86 (0.58, 1.29) |
| Individual THMs Mixture (Chloroform + Bromoform + Bromodichloromethane + Dibromochloromethane) + Adjust for Inorganics (Arsenic + Uranium + Nitrate-N) | 1.12 (0.97, 1.29) | 1.20 (1.03, 1.41) | 0.82 (0.57, 1.18) |
| Inorganics Mixture (Arsenic + Uranium + Nitrate-N) + Adjust for Individual THMs (Chloroform + Bromoform + Bromodichloromethane + Dibromochloromethane) | 1.06 (0.91, 1.23) | 1.06 (0.89, 1.25) | 1.00 (0.67, 1.47) |
| Inorganics Mixture (Arsenic + Uranium + Nitrate-N) + Adjust for TTHM | 0.99 (0.87, 1.12) | 0.98 (0.85, 1.13) | 0.94 (0.67, 1.31) |
| Inorganics Mixture (Arsenic + Uranium + Nitrate-N) | 0.96 (0.85, 1.09) | 0.95 (0.82, 1.08) | 0.99 (0.72, 1.36) |
| **Single contaminant adjusted for other contaminants, N=49,831** | | | |
| TTHM | 1.13 (0.99, 1.29) | 1.18 (1.02, 1.37) | 0.90 (0.65, 1.25) |
| Arsenic | 1.03 (0.93, 1.14) | 1.03 (0.92, 1.15) | 0.96 (0.74, 1.25) |
| Uranium | 0.95 (0.85, 1.06) | 0.93 (0.83, 1.05) | 1.13 (0.84, 1.51) |
| Nitrate-N | 1.00 (0.89, 1.13) | 1.03 (0.90, 1.17) | 0.85 (0.63, 1.15) |

[a]For contaminants included in the mixture, concentrations were log2 transformed and subsequently divided by the IQR. The effect of the drinking water contaminant mixture was evaluated using quantile q-computation, and hazards ratios are interpreted per IQR increase. Models were adjusted for contaminants not in the mixture (log2 transformed).

© 2026 Spaur M et al. JAMA Network Open.

[b]Concentrations were log2 transformed and subsequently divided by the IQR. Models were adjusted for all other contaminants (log2 transformed).
[c]Model was adjusted for baseline age (years) and $age^2$, BMI category (<25 $kg/m^2$, 25-<30 $kg/m^2$, >30 $kg/m^2$, or missing), and smoking status (never, former, current, or missing).

© 2026 Spaur M et al. JAMA Network Open.

**eTable 4. Hazard ratios (HR, 95% CI)s for uterine, endometrioid, and non-endometroid cancers per interquartile range (IQR) increase in the exposure mixture including haloacetic acids[a], and per single contaminants mutually adjusted[b]. Follow-up starts in 2010 and all averages represent 1990-2013 averages.**

| | Uterine Cancer | | Endometrioid Tumors | | Non-Endometrioid Tumors | |
|---|---|---|---|---|---|---|
| | **N Cases** | **HR (95% CI)[c]** | **N Cases** | **HR (95% CI)[c]** | **N Cases** | **HR (95% CI)[c]** |
| **Joint effect of mixture** | | | | | | |
| TTHM + Nitrate-N + Uranium + Arsenic + HAA5, N=32,344 | 322 | 1.24 (0.81, 1.89) | 247 | 1.11 (0.68, 1.80) | 70 | 1.93 (0.79, 4.72) |
| TTHM + Nitrate-N + Uranium + Arsenic + Individual HAAs, N=31,970 | 317 | 1.19 (0.73, 1.93) | 242 | 1.13 (0.65, 1.96) | 70 | 1.70 (0.60, 4.78) |
| **Single contaminant adjusted for other contaminants, N=32,344** | | | | | | |
| TTHM | 322 | 1.24 (1.06, 1.45) | 247 | 1.27 (1.06, 1.52) | 70 | 1.12 (0.79, 1.60) |
| HAA5 | 322 | 0.93 (0.76, 1.12) | 247 | 0.83 (0.66, 1.03) | 70 | 1.37 (0.88, 2.13) |
| Arsenic | 322 | 0.98 (0.82, 1.17) | 247 | 1.02 (0.83, 1.25) | 70 | 0.80 (0.54, 1.20) |
| Uranium | 322 | 1.03 (0.83, 1.28) | 247 | 1.01 (0.79, 1.30) | 70 | 1.19 (0.74, 1.93) |
| Nitrate-N | 322 | 0.97 (0.77, 1.22) | 247 | 0.90 (0.69, 1.18) | 70 | 1.21 (0.74, 1.97) |

[a]For contaminants included in the mixture, concentrations were log2 transformed and subsequently divided by the IQR. The effect of the drinking water contaminant mixture was evaluated using quantile q-computation, and hazards ratios are interpreted per IQR increase.
[b]Concentrations were log2 transformed and subsequently divided by the IQR. Models were adjusted for all other contaminants (log2 transformed).
[c]Model was adjusted for age in 2010 (years) and age$^2$, BMI category (<25 kg/m$^2$, 25-<30 kg/m$^2$, >30 kg/m$^2$, or missing), and smoking status (never, former, current, or missing).

© 2026 Spaur M et al. JAMA Network Open.

**eTable 5. Hazard Ratios (HR, 95% CIs) of uterine, endometrioid and non-endometrioid cancers per $\log_2$ increase in exposures[a,b], stratified by body mass index (BMI) category at enrollment.**

| BMI Category | N | Uterine Cancer N Cases | HR (95% CI)[c] | $P_{int}$[d] | Endometrioid Tumors N Cases | HR (95% CI)[c] | $P_{int}$[d] | Non-Endometrioid Tumors N Cases | HR (95% CI)[c] | $P_{int}$[d] |
|---|---|---|---|---|---|---|---|---|---|---|
| **TTHM (µg/L)** | | | | | | | | | | |
| <25 kg/m$^2$ | 31430 | 460 | 1.03 (1.00, 1.06) | | 388 | 1.04 (1.00, 1.07) | | 65 | 0.99 (0.91, 1.07) | |
| 25-<30 kg/m$^2$ | 12515 | 272 | 1.01 (0.97, 1.05) | | 222 | 1.00 (0.96, 1.04) | | 46 | 1.04 (0.95, 1.15) | |
| ≥30 kg/m$^2$ | 7168 | 270 | 1.02 (0.98, 1.06) | | 222 | 1.04 (0.99, 1.09) | | 39 | 0.95 (0.86, 1.04) | |
| **Chloroform (µg/L)** | | | | | | | | | | |
| <25 kg/m$^2$ | 31430 | 460 | 1.05 (1.01, 1.09) | | 388 | 1.07 (1.03, 1.11) | * | 65 | 0.95 (0.88, 1.04) | |
| 25-<30 kg/m$^2$ | 12515 | 272 | 1.01 (0.97, 1.06) | | 222 | 1.00 (0.96, 1.05) | | 46 | 1.07 (0.96, 1.19) | |
| ≥30 kg/m$^2$ | 7168 | 270 | 1.02 (0.98, 1.06) | | 222 | 1.04 (0.99, 1.09) | | 39 | 0.95 (0.85, 1.06) | |
| **Bromoform (µg/L)** | | | | | | | | | | |
| <25 kg/m$^2$ | 31430 | 460 | 1.00 (0.96, 1.04) | | 388 | 1.00 (0.95, 1.04) | | 65 | 1.00 (0.90, 1.12) | |
| 25-<30 kg/m$^2$ | 12515 | 272 | 1.00 (0.95, 1.06) | | 222 | 1.02 (0.96, 1.08) | | 46 | 0.93 (0.81, 1.06) | |
| ≥30 kg/m$^2$ | 7168 | 270 | 1.02 (0.97, 1.08) | | 222 | 1.05 (0.99, 1.11) | | 39 | 0.93 (0.80, 1.08) | |
| **Bromodichloromethane (µg/L)** | | | | | | | | | | |
| <25 kg/m$^2$ | 31430 | 460 | 1.03 (0.99, 1.07) | | 388 | 1.05 (1.01, 1.09) | | 65 | 0.96 (0.87, 1.05) | |
| 25-<30 kg/m$^2$ | 12515 | 272 | 1.02 (0.97, 1.07) | | 222 | 1.02 (0.97, 1.07) | | 46 | 1.04 (0.93, 1.17) | |
| ≥30 kg/m$^2$ | 7168 | 270 | 1.03 (0.98, 1.08) | | 222 | 1.05 (1.00, 1.11) | | 39 | 0.93 (0.82, 1.05) | |
| **Dibromochloromethane (µg/L)** | | | | | | | | | | |
| <25 kg/m$^2$ | 31430 | 460 | 1.01 (0.98, 1.05) | | 388 | 1.02 (0.98, 1.06) | | 65 | 0.99 (0.90, 1.08) | |
| 25-<30 kg/m$^2$ | 12515 | 272 | 1.02 (0.97, 1.06) | | 222 | 1.02 (0.97, 1.07) | | 46 | 1.00 (0.90, 1.12) | |
| ≥30 kg/m$^2$ | 7168 | 270 | 1.02 (0.97, 1.06) | | 222 | 1.04 (0.99, 1.10) | | 39 | 0.90 (0.79, 1.02) | |
| **Arsenic (µg/L)** | | | | | | | | | | |
| <25 kg/m$^2$ | 31398 | 460 | 0.96 (0.88, 1.03) | | 388 | 0.94 (0.86, 1.03) | | 65 | 1.02 (0.83, 1.26) | |
| 25-<30 kg/m$^2$ | 12513 | 272 | 1.01 (0.92, 1.12) | | 222 | 1.03 (0.93, 1.16) | | 46 | 0.95 (0.74, 1.21) | |
| ≥30 kg/m$^2$ | 7162 | 269 | 0.97 (0.88, 1.07) | | 221 | 0.93 (0.83, 1.04) | | 39 | 1.02 (0.79, 1.33) | |
| **Uranium (µg/L)** | | | | | | | | | | |
| <25 kg/m$^2$ | 29446 | 435 | 0.96 (0.90, 1.02) | | 365 | 0.95 (0.89, 1.02) | | 64 | 1.03 (0.87, 1.21) | |
| 25-<30 kg/m$^2$ | 11767 | 251 | 0.99 (0.91, 1.07) | | 204 | 0.99 (0.91, 1.09) | | 43 | 0.95 (0.78, 1.15) | |
| ≥30 kg/m$^2$ | 6747 | 256 | 0.96 (0.89, 1.05) | | 209 | 0.93 (0.86, 1.02) | | 39 | 1.12 (0.91, 1.39) | |
| **Nitrate-N (mg/L)** | | | | | | | | | | |

© 2026 Spaur M et al. JAMA Network Open.

| | | | | | | | | |
|---|---|---|---|---|---|---|---|---|
| <25 kg/m$^2$ | 31409 | 460 | 0.97 (0.93, 1.02) | 388 | 0.97 (0.93, 1.02) | * | 65 | 0.95 (0.85, 1.07) |
| 25-<30 kg/m$^2$ | 12513 | 272 | 0.98 (0.92, 1.04) | 222 | 0.98 (0.92, 1.04) | | 46 | 0.98 (0.85, 1.13) |
| ≥30 kg/m$^2$ | 7164 | 269 | 0.99 (0.93, 1.05) | 221 | 0.98 (0.92, 1.05) | | 39 | 1.04 (0.89, 1.22) |

[a]Community water system (CWS) exposures were linked to the address at enrollment and represent 1990-2005 average concentrations of individual and total trihalomethanes (TTHM), arsenic, uranium, and nitrate-N.
[b]Analyses were restricted to participants with a residential duration at enrollment ≥ 10 years.
[c]Model was adjusted for baseline age (years) and age$^2$, and smoking status (never, former, current, or missing).
[d]P value for interaction was obtained using the Chi squared likelihood ratio test: P<0.05: *, P<0.01: **, P<0.001: ***. Statistical interaction by BMI was observed for chloroform ($p_{interaction}$ = 0.03) and nitrate ($p_{interaction}$ = 0.01) for endometrioid tumor risk only (p<0.05). No statistical interaction was observed in all other analyses.

© 2026 Spaur M et al. JAMA Network Open.

**eTable 6. Hazard Ratios (95% CIs) of uterine, endometrioid and non-endometrioid cancers per $log_2$ increase in exposures[a,b], stratified by menopausal status[c] at enrollment.**

| Menopause status | N | Uterine Cancer | | Endometrioid Tumors | | Non-Endometrioid Tumors | |
|---|---|---|---|---|---|---|---|
| | | N Cases | HR (95% CI)[d] | N Cases | HR (95% CI)[d] | N Cases | HR (95% CI)[d] |
| **TTHM (μg/L), N=53,100** | | | | | | | |
| Pre, N | 28062 | 422 | 1.04 (1.01, 1.07) | 348 | 1.04 (1.01, 1.08) | 62 | 1.03 (0.95, 1.12) |
| Peri/ Post | 25038 | 616 | 1.01 (0.98, 1.03) | 516 | 1.01 (0.98, 1.04) | 92 | 0.98 (0.92, 1.04) |
| **Chloroform (μg/L), N=53,100** | | | | | | | |
| Pre | 28062 | 422 | 1.04 (1.01, 1.08) | 348 | 1.05 (1.01, 1.09) | 62 | 1.03 (0.94, 1.12) |
| Peri/Post | 25038 | 616 | 1.01 (0.99, 1.04) | 516 | 1.02 (0.99, 1.06) | 92 | 0.96 (0.89, 1.03) |
| **Bromoform (μg/L), N=53,100** | | | | | | | |
| Pre | 28062 | 422 | 1.03 (0.98, 1.07) | 348 | 1.04 (0.99, 1.09) | 62 | 1.02 (0.91, 1.14) |
| Peri/Post | 25038 | 616 | 1.00 (0.96, 1.03) | 516 | 1.00 (0.97, 1.04) | 92 | 0.94 (0.86, 1.04) |
| **Bromodichloromethane (μg/L), N=53,100** | | | | | | | |
| Pre | 28062 | 422 | 1.04 (1.00, 1.08) | 348 | 1.05 (1.00, 1.09) | 62 | 1.02 (0.93, 1.13) |
| Peri/Post | 25038 | 616 | 1.01 (0.98, 1.05) | 516 | 1.03 (0.99, 1.06) | 92 | 0.96 (0.88, 1.03) |
| **Dibromochloromethane (μg/L), N=53,100** | | | | | | | |
| Pre | 28062 | 422 | 1.02 (0.98, 1.06) | 348 | 1.03 (0.99, 1.07) | 62 | 1.01 (0.92, 1.11) |
| Peri/Post | 25038 | 616 | 1.01 (0.98, 1.04) | 516 | 1.02 (0.98, 1.05) | 92 | 0.96 (0.89, 1.04) |
| **Arsenic (μg/L), N=53,060** | | | | | | | |
| Pre | 28033 | 421 | 1.02 (0.94, 1.10) | 347 | 1.00 (0.92, 1.10) | 62 | 0.99 (0.81, 1.23) |
| Peri/Post | 25027 | 616 | 0.97 (0.90, 1.03) | 516 | 0.96 (0.89, 1.03) | 92 | 1.01 (0.85, 1.20) |
| **Uranium (μg/L), N=49,831** | | | | | | | |
| Pre | 26253 | 394 | 1.00 (0.94, 1.07) | 323 | 0.99 (0.92, 1.06) | 60 | 1.13 (0.95, 1.35) |
| Peri/Post | 23578 | 580 | 0.96 (0.91, 1.01) | 484 | 0.96 (0.90, 1.01) | 89 | 0.97 (0.85, 1.11) |
| **Nitrate-N (mg/L), N=53,073** | | | | | | | |
| Pre | 28042 | 421 | 1.00 (0.95, 1.05) | 347 | 1.00 (0.95, 1.06) | 62 | 0.97 (0.86, 1.09) |
| Peri/Post | 25031 | 616 | 0.98 (0.95, 1.02) | 516 | 0.98 (0.94, 1.02) | 92 | 0.98 (0.89, 1.08) |

[a]Community water system (CWS) exposures were linked to the address at enrollment and represent 1990-2005 average concentrations of individual and total trihalomethanes (TTHM), arsenic, uranium, and nitrate-N.

[b]Analyses were restricted to participants with a residential duration at enrollment ≥ 10 years.

[c]Women who were missing menopause status at baseline (N=3,449) were categorized as peri/post-menopausal if they were over 52 years of age at baseline, and as pre-menopausal if they were <= 52 years of age at baseline. No statistical interaction by menopausal status was observed in all analyses, using the Chi squared likelihood ratio test ($P_{int}$>0.05).

[d]Model was adjusted for baseline age (years) and age$^2$, BMI category (<25 kg/m$^2$, 25-<30 kg/m$^2$, >30 kg/m$^2$, or missing), and smoking status (never, former, current, or missing).

© 2026 Spaur M et al. JAMA Network Open.

**eTable 7. Hazard Ratios (HR, 95% CIs) of uterine, endometrioid and non-endometrioid cancers per $log_2$ increase in exposures[a,b], stratified by smoking status[c] at enrollment.**

| | | Uterine Cancer | | Endometrioid Tumors | | Non-Endometrioid Tumors | |
|---|---|---|---|---|---|---|---|
| **Smoking status** | **N** | **N Cases** | **HR (95% CI)[d]** | **N Cases** | **HR (95% CI)[d]** | **N Cases** | **HR (95% CI)[d]** |
| **TTHM (µg/L)** | | | | | | | |
| Never | 35484 | 693 | 1.02 (1.00, 1.05) | 570 | 1.03 (1.00, 1.06) | 112 | 1.01 (0.95, 1.07) |
| Former | 14659 | 303 | 1.02 (0.98, 1.06) | 262 | 1.03 (0.99, 1.07) | 36 | 0.93 (0.84, 1.03) |
| Current | 2645 | 39 | 1.00 (0.90, 1.11) | 29 | 0.94 (0.84, 1.06) | 6 | 1.54 (0.91, 2.61) |
| **Chloroform (µg/L)** | | | | | | | |
| Never | 35484 | 693 | 1.03 (1.01, 1.06) | 570 | 1.04 (1.01, 1.07) | 112 | 0.99 (0.93, 1.06) |
| Former | 14659 | 303 | 1.01 (0.97, 1.06) | 262 | 1.03 (0.98, 1.07) | 36 | 0.93 (0.83, 1.04) |
| Current | 2645 | 39 | 1.04 (0.93, 1.17) | 29 | 0.99 (0.87, 1.13) | 6 | 1.53 (0.99, 2.34) |
| **Bromoform (µg/L)** | | | | | | | |
| Never | 35484 | 693 | 1.01 (0.98, 1.05) | 570 | 1.02 (0.98, 1.05) | 112 | 1.00 (0.92, 1.09) |
| Former | 14659 | 303 | 1.01 (0.96, 1.06) | 262 | 1.04 (0.98, 1.09) | 36 | 0.86 (0.74, 1.01) |
| Current | 2645 | 39 | 0.95 (0.82, 1.10) | 29 | 0.93 (0.79, 1.11) | 6 | 1.06 (0.74, 1.52) |
| **Bromodichloromethane (µg/L)** | | | | | | | |
| Never | 35484 | 693 | 1.03 (1.00, 1.06) | 570 | 1.04 (1.01, 1.08) | 112 | 0.99 (0.92, 1.07) |
| Former | 14659 | 303 | 1.00 (0.96, 1.05) | 262 | 1.02 (0.98, 1.07) | 36 | 0.91 (0.80, 1.03) |
| Current | 2645 | 39 | 1.01 (0.90, 1.15) | 29 | 0.98 (0.85, 1.14) | 6 | 1.36 (0.91, 2.03) |
| **Dibromochloromethane (µg/L)** | | | | | | | |
| Never | 35484 | 693 | 1.02 (0.99, 1.05) | 570 | 1.03 (1.00, 1.06) | 112 | 1.00 (0.93, 1.07) |
| Former | 14659 | 303 | 1.00 (0.96, 1.05) | 262 | 1.03 (0.98, 1.07) | 36 | 0.88 (0.78, 1.01) |
| Current | 2645 | 39 | 1.00 (0.89, 1.12) | 29 | 0.98 (0.86, 1.13) | 6 | 1.22 (0.88, 1.70) |
| **Arsenic (µg/L)** | | | | | | | |
| Never | 35457 | 692 | 0.97 (0.91, 1.03) | 569 | 0.96 (0.90, 1.03) | 112 | 0.96 (0.82, 1.12) |
| Former | 14650 | 303 | 1.03 (0.93, 1.13) | 262 | 1.01 (0.91, 1.12) | 36 | 1.16 (0.89, 1.52) |
| Current | 2642 | 39 | 0.93 (0.71, 1.23) | 29 | 0.99 (0.73, 1.36) | 6 | 0.73 (0.33, 1.57) |
| **Uranium (µg/L)** | | | | | | | |
| Never | 33276 | 649 | 0.98 (0.93, 1.03) | 532 | 0.96 (0.91, 1.02) | 107 | 1.07 (0.94, 1.22) |
| Former | 13766 | 286 | 0.98 (0.91, 1.06) | 246 | 0.99 (0.91, 1.07) | 36 | 0.97 (0.79, 1.20) |
| Current | 2492 | 37 | 0.89 (0.72, 1.10) | 27 | 0.91 (0.71, 1.17) | 6 | 0.71 (0.42, 1.21) |
| **Nitrate-N (mg/L)** | | | | | | | |
| Never | 35466 | 692 | 0.99 (0.96, 1.03) | 569 | 0.99 (0.95, 1.03) | 112 | 1.01 (0.92, 1.11) |
| Former | 14651 | 303 | 0.98 (0.93, 1.04) | 262 | 1.00 (0.94, 1.06) | 36 | 0.88 (0.74, 1.03) |

© 2026 Spaur M et al. JAMA Network Open.

| | | | | | | |
|---|---|---|---|---|---|---|
| Current | 2645 | 39 | 0.91 (0.78, 1.07) | 29 | 0.92 (0.76, 1.10) | 6 | 0.75 (0.50, 1.12) |

[a]Community water system (CWS) exposures were linked to the address at enrollment and represent 1990-2005 average concentrations of individual and total trihalomethanes (TTHM), arsenic, uranium, and nitrate-N.
[b]Analyses were restricted to participants with a residential duration at enrollment $\geq$ 10 years.
[c]No statistical interaction by smoking status was observed in all analyses, using the Chi squared likelihood ratio test ($P_{int}$>0.05).
[d]Model was adjusted for baseline age (years) and age$^2$, and BMI category (<25 kg/m$^2$, 25-<30 kg/m$^2$, >30 kg/m$^2$, or missing).

© 2026 Spaur M et al. JAMA Network Open.

**eTable 8. Hazard Ratios (HR, 95% CIs) of uterine, endometrioid and non-endometrioid cancers per $\log_2$ increase in exposures[a,b], stratified by hormone therapy (HT) use at enrollment[c].**

| | | Uterine Cancer | | Endometrioid Tumors | | Non-Endometrioid Tumors | |
|---|---|---|---|---|---|---|---|
| **HT Use** | **N** | **N cases** | **HR (95% CI)[d]** | **N Cases** | **HR (95% CI)[d]** | **N cases** | **HR (95% CI)[d]** |
| **TTHM (µg/L)** | | | | | | | |
| Never | 7483 | 158 | 1.01 (0.96, 1.06) | 120 | 1.01 (0.95, 1.07) | 35 | 1.00 (0.89, 1.11) |
| Ever | 17567 | 442 | 1.01 (0.98, 1.05) | 383 | 1.02 (0.99, 1.05) | 50 | 0.98 (0.90, 1.07) |
| **Chloroform (µg/L)** | | | | | | | |
| Never | 7483 | 158 | 0.98 (0.93, 1.04) | 120 | 0.99 (0.93, 1.06) | 35 | 0.96 (0.85, 1.08) |
| Ever | 17567 | 442 | 1.03 (0.99, 1.06) | 383 | 1.04 (1.00, 1.08) | 50 | 0.98 (0.89, 1.08) |
| **Bromoform (µg/L)** | | | | | | | |
| Never | 7483 | 158 | 1.00 (0.93, 1.07) | 120 | 1.00 (0.92, 1.08) | 35 | 0.99 (0.85, 1.15) |
| Ever | 17567 | 442 | 0.98 (0.94, 1.02) | 383 | 0.99 (0.95, 1.04) | 50 | 0.91 (0.80, 1.03) |
| **Bromodichloromethane (µg/L)** | | | | | | | |
| Never Use | 7483 | 158 | 1.01 (0.95, 1.07) | 120 | 1.00 (0.94, 1.07) | 35 | 1.01 (0.89, 1.15) |
| Ever Use | 17567 | 442 | 1.01 (0.97, 1.05) | 383 | 1.02 (0.98, 1.06) | 50 | 0.94 (0.85, 1.05) |
| **Dibromochloromethane (µg/L)** | | | | | | | |
| Never | 7483 | 158 | 1.01 (0.95, 1.08) | 120 | 1.01 (0.94, 1.08) | 35 | 1.01 (0.89, 1.15) |
| Ever | 17567 | 442 | 1.00 (0.96, 1.03) | 383 | 1.01 (0.97, 1.05) | 50 | 0.93 (0.84, 1.04) |
| **Arsenic (µg/L)** | | | | | | | |
| Never | 7479 | 158 | 0.95 (0.83, 1.09) | 120 | 0.92 (0.78, 1.08) | 35 | 1.11 (0.84, 1.46) |
| Ever | 17559 | 442 | 0.97 (0.90, 1.06) | 383 | 0.97 (0.89, 1.06) | 50 | 1.00 (0.78, 1.26) |
| **Uranium (µg/L)** | | | | | | | |
| Never | 7069 | 156 | 0.97 (0.88, 1.08) | 120 | 0.97 (0.86, 1.09) | 34 | 0.97 (0.78, 1.20) |
| Ever | 16517 | 410 | 0.95 (0.89, 1.01) | 352 | 0.93 (0.87, 1.00) | 49 | 1.06 (0.88, 1.28) |
| **Nitrate-N (mg/L)** | | | | | | | |
| Never | 7480 | 158 | 1.01 (0.93, 1.09) | 120 | 1.03 (0.94, 1.12) | 35 | 0.93 (0.79, 1.10) |
| Ever | 17562 | 442 | 0.97 (0.93, 1.02) | 383 | 0.96 (0.91, 1.01) | 50 | 1.03 (0.90, 1.19) |

[a]Community water system (CWS) exposures were linked to the address at enrollment and represent 1990-2005 average concentrations of individual and total trihalomethanes (TTHM), arsenic, uranium, and nitrate-N.
[b]Analyses were restricted to participants with a residential duration at enrollment ≥ 10 years.
[c]N=25,495 women who were pre-menopausal at baseline were considered to never use HT and were excluded from this analysis. N=2,555 women who were peri/post-menopausal and were missing HT use information. No statistical interaction by HT use was observed in all analyses, using the Chi squared likelihood ratio test ($P_{int}$>0.05).
[d]Model was adjusted for baseline age (years) and age$^2$, BMI category (<25 kg/m2, 25-<30 kg/m2, >30 kg/m2, or missing), and smoking status (never, former, current, or missing).

© 2026 Spaur M et al. JAMA Network Open.

**eTable 9. Hazard Ratios (HR, 95% CIs) of uterine, endometrioid and non-endometrioid cancer per doubling and tertiles of time-varying exposures, lagged 5 years[a].**

| | Uterine Cancer | | Endometrioid Tumors | | Non-Endometrioid Tumors | |
|---|---|---|---|---|---|---|
| **Concentration Range** | **N Cases** | **HR (95% CI)[b]** | **N Cases** | **HR (95% CI)[b]** | **N Cases** | **HR (95% CI)[b]** |
| **TTHM (µg/L)**, N=76774, total person-years=1431033 | | | | | | |
| Log2 | 1335 | 1.02 (1.00, 1.04) | 1112 | 1.02 (1.00, 1.04) | 197 | 1.02 (0.96, 1.08) |
| T1 (0,3.14) | 395 | 1.0 (reference) | 343 | 1.0 (reference) | 45 | 1.0 (reference) |
| T2 (3.14,16.64) | 449 | 1.05 (0.92, 1.21) | 357 | 0.98 (0.84, 1.14) | 81 | 1.49 (1.03, 2.16) |
| T3 (16.64,172.05) | 491 | 1.13 (0.99, 1.29) | 412 | 1.11 (0.96, 1.29) | 71 | 1.26 (0.86, 1.83) |
| P trend[c] | | 0.25 | | 0.24 | | 0.76 |
| **Chloroform (µg/L)**, N=76782, total person-years=1431142 | | | | | | |
| Log2 | 1336 | 1.02 (1.00, 1.04) | 1112 | 1.02 (1.00, 1.05) | 198 | 1.01 (0.96, 1.06) |
| T1 (0,0.55) | 390 | 1.0 (reference) | 326 | 1.0 (reference) | 56 | 1.0 (reference) |
| T2 (0.55,5.77) | 455 | 1.09 (0.95, 1.25) | 377 | 1.10 (0.95, 1.28) | 71 | 1.03 (0.72, 1.47) |
| T3 (5.77,165) | 491 | 1.15 (1.01, 1.32) | 409 | 1.18 (1.01, 1.36) | 71 | 1.01 (0.71, 1.44) |
| P trend[c] | | 0.16 | | 0.13 | | 0.82 |
| **Bromoform (µg/L)**, N=76782, total person-years=1431142 | | | | | | |
| Log2 | 1336 | 1.00 (0.98, 1.03) | 1112 | 1.00 (0.98, 1.03) | 198 | 1.00 (0.94, 1.07) |
| T1 (0,0.10) | 421 | 1.0 (reference) | 350 | 1.0 (reference) | 62 | 1.0 (reference) |
| T2 (0.10,1.03) | 456 | 1.03 (0.90, 1.18) | 390 | 1.08 (0.93, 1.24) | 56 | 0.79 (0.55, 1.14) |
| T3 (1.03,130) | 459 | 1.03 (0.90, 1.18) | 372 | 1.02 (0.88, 1.19) | 80 | 1.09 (0.78, 1.52) |
| P trend[c] | | 0.98 | | 0.68 | | 0.24 |
| **Bromodichloromethane (µg/L)**, N=76782, total person-years=1431142 | | | | | | |
| Log2 | 1336 | 1.02 (1.00, 1.04) | 1112 | 1.02 (1.00, 1.04) | 198 | 1.02 (0.97, 1.08) |
| T1 (0,0.29) | 397 | 1.0 (reference) | 338 | 1.0 (reference) | 49 | 1.0 (reference) |
| T2 (0.29,3.65) | 463 | 1.08 (0.94, 1.24) | 377 | 1.06 (0.91, 1.23) | 78 | 1.27 (0.89, 1.83) |
| T3 (3.65,120) | 476 | 1.09 (0.95, 1.25) | 397 | 1.10 (0.95, 1.27) | 71 | 1.12 (0.77, 1.62) |
| P trend[c] | | 0.68 | | 0.52 | | 0.79 |
| **Dibromochloromethane (µg/L)**, N=76782, total person-years=1431142 | | | | | | |
| Log2 | 1336 | 1.01 (0.99, 1.03) | 1112 | 1.01 (0.99, 1.03) | 198 | 1.02 (0.96, 1.07) |
| T1 (0,0.18) | 409 | 1.0 (reference) | 344 | 1.0 (reference) | 53 | 1.0 (reference) |
| T2 (0.18,2.32) | 460 | 1.03 (0.90, 1.18) | 384 | 1.05 (0.91, 1.22) | 71 | 1.07 (0.75, 1.54) |
| T3 (2.32,77.50) | 467 | 1.06 (0.92, 1.21) | 384 | 1.06 (0.92, 1.23) | 74 | 1.11 (0.78, 1.59) |
| P trend[c] | | 0.72 | | 0.79 | | 0.73 |
| **Arsenic (µg/L)**, N=75579, total person-years=1407655 | | | | | | |
| Log2 | 1319 | 0.96 (0.92, 1.00) | 1097 | 0.96 (0.92, 1.00) | 196 | 0.94 (0.84, 1.06) |
| T1 (0,0.72) | 464 | 1.0 (reference) | 388 | 1.0 (reference) | 69 | 1.0 (reference) |
| T2 (0.72,1.45) | 426 | 0.93 (0.82, 1.06) | 354 | 0.94 (0.81, 1.08) | 62 | 0.86 (0.61, 1.22) |
| T3 (1.45,100.0) | 429 | 0.95 (0.83, 1.08) | 355 | 0.95 (0.82, 1.10) | 65 | 0.91 (0.65, 1.28) |
| P trend[c] | | 0.79 | | 0.78 | | 0.83 |
| **Uranium (µg/L)**, N=69503, total person-years=1288873 | | | | | | |
| Log2 | 1217 | 0.97 (0.94, 1.01) | 1010 | 0.97 (0.93, 1.01) | 184 | 0.99 (0.90, 1.09) |
| T1 (0.09,3.12) | 436 | 1.0 (reference) | 364 | 1.0 (reference) | 63 | 1.0 (reference) |
| T2 (3.12,5.70) | 397 | 1.00 (0.87, 1.15) | 328 | 0.98 (0.84, 1.14) | 62 | 1.14 (0.8, 1.62) |
| T3 (5.70,101.62) | 384 | 0.92 (0.81, 1.06) | 318 | 0.91 (0.78, 1.05) | 59 | 1.05 (0.74, 1.50) |
| P trend[c] | | 0.35 | | 0.26 | | 0.70 |
| **Nitrate-N (mg/L)**, N=75699, total person-years=1409876 | | | | | | |
| Log2 | 1322 | 0.98 (0.95, 1.00) | 1099 | 0.98 (0.95, 1.01) | 197 | 0.96 (0.90, 1.03) |

© 2026 Spaur M et al. JAMA Network Open.

| | | | | | | |
|---|---|---|---|---|---|---|
| T1 (0.01,0.28) | 462 | 1.0 (reference) | 384 | 1.0 (reference) | 69 | 1.0 (reference) |
| T2 (0.28,1.42) | 455 | 0.97 (0.85, 1.10) | 378 | 0.97 (0.84, 1.12) | 68 | 0.94 (0.67, 1.32) |
| T3 (1.42,19.22) | 405 | 0.87 (0.76, 10) | 337 | 0.88 (0.76, 1.02) | 60 | 0.85 (0.60, 1.20) |
| P trend[c] | | 0.16 | | 0.22 | | 0.53 |

[a]Community water system (CWS) exposures were linked to address(es) from the residential history and represent time-varying cumulative average exposures, lagged 5 years.
[b]Model was adjusted for age (time varying variable) and age$^2$, BMI category (<25 kg/m$^2$, 25-<30 kg/m$^2$, >30 kg/m$^2$, or missing), and smoking status (never, former, current, or missing).
[c]P trend was evaluated using the median of each tertile.

© 2026 Spaur M et al. JAMA Network Open.

**eTable 10. Hazard Ratios (HR, 95% CIs) of uterine, endometrioid and non-endometrioid cancer per doubling and tertiles of time-varying cumulative average exposures, lagged 5 years among the main analytic population[a].**

| | Uterine Cancer | | Endometrioid Tumors | | Non-Endometrioid Tumors | |
|---|---|---|---|---|---|---|
| **Concentration Range** | **N Cases** | **HR (95% CI)[b]** | **N Cases** | **HR (95% CI)[b]** | **N Cases** | **HR (95% CI)[b]** |
| **TTHM (µg/L), N=**51624, total person-years=1002013 | | | | | | |
| Log2 | 1012 | 1.01 (0.98, 1.03) | 842 | 1.01 (0.99, 1.04) | 150 | 0.99 (0.93, 1.06) |
| T1 (0,2.72) | 309 | 1.0 (ref) | 262 | 1.0 (ref) | 40 | 1.0 (ref) |
| T2 (2.72,16.24) | 334 | 0.99 (0.84, 1.15) | 268 | 0.95 (0.80, 1.13) | 57 | 1.19 (0.79, 1.78) |
| T3 (16.24,172.05) | 369 | 1.08 (0.93, 1.26) | 312 | 1.10 (0.93, 1.30) | 53 | 1.06 (0.70, 1.60) |
| P trend[c] | | 0.42 | | 0.23 | | 0.80 |
| **Chloroform (µg/L), N=**51626, total person-years=1002045 | | | | | | |
| Log2 | 1013 | 1.02 (1.00, 1.04) | 842 | 1.03 (1.00, 1.05) | 151 | 0.99 (0.93, 1.05) |
| T1 (0,0.51) | 301 | 1.0 (ref) | 247 | 1.0 (ref) | 47 | 1.0 (ref) |
| T2 (0.51,5.55) | 338 | 1.04 (0.89, 1.22) | 279 | 1.07 (0.90, 1.28) | 53 | 0.91 (0.61, 1.35) |
| T3 (5.55,165.0) | 374 | 1.14 (0.97, 1.33) | 316 | 1.20 (1.01, 1.42) | 51 | 0.86 (0.57, 1.28) |
| P trend[c] | | 0.19 | | 0.07 | | 0.38 |
| **Bromoform (µg/L), N=**51626, total person-years=1002045 | | | | | | |
| Log2 | 1013 | 1.00 (0.97, 1.02) | 842 | 1.00 (0.97, 1.03) | 151 | 0.97 (0.91, 1.05) |
| T1 (0,0.10) | 325 | 1.0 (ref) | 268 | 1.0 (ref) | 50 | 1.0 (ref) |
| T2 (0.10,0.96) | 343 | 1.00 (0.86, 1.17) | 291 | 1.04 (0.88, 1.23) | 43 | 0.76 (0.50, 1.14) |
| T3 (0.96,15.77) | 345 | 1.00 (0.86, 1.16) | 283 | 1.01 (0.86, 1.20) | 58 | 0.98 (0.67, 1.43) |
| P trend[c] | | 0.77 | | 0.74 | | 0.61 |
| **Bromodichloromethane, N=**51626, total person-years=1002045 | | | | | | |
| Log2 | 1013 | 1.02 (0.99, 1.04) | 842 | 1.02 (1.00, 1.05) | 151 | 1.00 (0.94, 1.07) |
| T1 (0,0.27) | 301 | 1.0 (ref) | 253 | 1.0 (ref) | 40 | 1.0 (ref) |
| T2 (0.27,3.37) | 355 | 1.09 (0.94, 1.28) | 287 | 1.08 (0.91, 1.28) | 61 | 1.22 (0.81, 1.83) |
| T3 (3.37,31.0) | 357 | 1.08 (0.92, 1.26) | 302 | 1.12 (0.94, 1.32) | 50 | 0.96 (0.63, 1.46) |
| P trend[c] | | 0.87 | | 0.45 | | 0.32 |
| **Dibromochloromethane (µg/L), N=**51626, total person-years=1002045 | | | | | | |
| Log2 | 1013 | 1.01 (0.98, 1.03) | 842 | 1.01 (0.99, 1.04) | 151 | 0.99 (0.93, 1.05) |
| T1 (0,0.16) | 309 | 1.0 (ref) | 257 | 1.0 (ref) | 41 | 1.0 (ref) |
| T2 (0.16,2.12) | 349 | 1.04 (0.89, 1.22) | 294 | 1.08 (0.92, 1.28) | 53 | 1.04 (0.69, 1.56) |
| T3 (2.12,38.0) | 355 | 1.06 (0.91, 1.23) | 291 | 1.07 (0.90, 1.27) | 57 | 1.10 (0.73, 1.66) |
| P trend[c] | | 0.83 | | 0.89 | | 0.75 |
| **Arsenic (µg/L), N=**51114, total person-years=991634 | | | | | | |
| Log2 | 1006 | 0.96 (0.91, 1.01) | 837 | 0.95 (0.9, 1) | 149 | 0.98 (0.86, 1.12) |
| T1 (0,0.67) | 361 | 1.0 (ref) | 304 | 1.0 (ref) | 53 | 1.0 (ref) |
| T2 (0.67,1.48) | 315 | 0.87 (0.75, 1.01) | 261 | 0.86 (0.73, 1.02) | 46 | 0.84 (0.57, 1.25) |
| T3 (1.48,100.0) | 330 | 0.92 (0.79, 1.07) | 272 | 0.91 (0.77, 1.07) | 50 | 0.89 (0.60, 1.31) |
| P trend[c] | | 0.66 | | 0.60 | | 0.81 |
| **Uranium (µg/L), N=**46924, total person-years=908819 | | | | | | |
| Log2 | 921 | 0.97 (0.93, 1.02) | 763 | 0.97 (0.92, 1.01) | 141 | 1.02 (0.91, 1.13) |
| T1 (0.09,2.90) | 334 | 1.0 (ref) | 280 | 1.0 (ref) | 46 | 1.0 (ref) |
| T2 (2.90,5.72) | 286 | 0.91 (0.78, 1.07) | 236 | 0.90 (0.75, 1.07) | 47 | 1.12 (0.74, 1.68) |
| T3 (5.72,101.62) | 301 | 0.93 (0.80, 1.09) | 247 | 0.90 (0.76, 1.07) | 48 | 1.14 (0.76, 1.72) |
| P trend[c] | | 0.5 | | 0.3 | | 0.4 |

© 2026 Spaur M et al. JAMA Network Open.

| | | | | | | |
|---|---|---|---|---|---|---|
| **Nitrate-N (mg/L), N=**51184, total person-years=993009 | | | | | | |
| Log2 | 1007 | 0.98 (0.95, 1.01) | 837 | 0.98 (0.95, 1.01) | 150 | 0.97 (0.90, 1.05) |
| T1 (0.01,0.26) | 349 | 1.0 (ref) | 288 | 1.0 (ref) | 56 | 1.0 (ref) |
| T2 (0.26,1.52) | 340 | 0.96 (0.83, 1.12) | 286 | 0.98 (0.84, 1.16) | 45 | 0.78 (0.52, 1.15) |
| T3 (1.52,19.22) | 318 | 0.9 (0.77, 1.05) | 263 | 0.91 (0.77, 1.07) | 49 | 0.84 (0.57, 1.23) |
| P trend[c] | | 0.42 | | 0.43 | | 0.85 |

[a]Community water system (CWS) exposures were linked to address(es) from the residential history and represent time-varying cumulative average exposures, lagged 5 years. Analyses were limited to the main analytic population.
[b]Model was adjusted for age (time varying variable) and age$^2$, BMI category (<25 kg/m$^2$, 25-<30 kg/m$^2$, >30 kg/m$^2$, or missing), and smoking status (never, former, current, or missing).
[c]P trend was evaluated using the median of each tertile.

© 2026 Spaur M et al. JAMA Network Open.

**eTable 11. Hazard Ratios (HR, 95% CIs) of uterine, endometrioid and non-endometrioid cancer per log$_2$ increase and tertiles of time-varying cumulative average exposures, lagged 10 years[a].**

| | Uterine Cancer | | Endometrioid Tumors | | Non-Endometrioid Tumors | |
|---|---|---|---|---|---|---|
| **Concentration Range** | **N Cases** | **HR (95% CI)[b]** | **N Cases** | **HR (95% CI)[b]** | **N Cases** | **HR (95% CI)[b]** |
| **TTHM (µg/L), N=**70727, total person-years=1136643 | | | | | | |
| Log2 | 1082 | 1.02 (1.00, 1.04) | 881 | 1.02 (1.00, 1.05) | 180 | 1.01 (0.96, 1.07) |
| T1 (0,2.49) | 334 | 1.0 (reference) | 281 | 1.0 (reference) | 48 | 1.0 (reference) |
| T2 (2.49,15.44) | 348 | 1.02 (0.88, 1.19) | 268 | 0.95 (0.80, 1.13) | 70 | 1.31 (0.91, 1.91) |
| T3 (15.44,172.05) | 400 | 1.15 (0.99, 1.33) | 332 | 1.16 (0.98, 1.36) | 62 | 1.11 (0.76, 1.63) |
| P trend[c] | | 0.17 | | 0.10 | | 0.90 |
| **Chloroform (µg/L), N=**70734, total person-years=1136722 | | | | | | |
| Log2 | 1083 | 1.03 (1.00, 1.05) | 881 | 1.03 (1.00, 1.05) | 181 | 1.00 (0.95, 1.06) |
| T1 (0,0.43) | 324 | 1.0 (reference) | 266 | 1.0 (reference) | 52 | 1.0 (reference) |
| T2 (0.43,5.41) | 362 | 1.08 (0.93, 1.25) | 290 | 1.07 (0.91, 1.27) | 66 | 1.11 (0.77, 1.60) |
| T3 (5.41,165) | 397 | 1.18 (1.01, 1.36) | 325 | 1.20 (1.02, 1.41) | 63 | 1.04 (0.72, 1.51) |
| P trend[c] | | 0.13 | | 0.10 | | 0.86 |
| **Bromoform (µg/L), N=**70734, total person-years=1136722 | | | | | | |
| Log2 | 1083 | 1.00 (0.98, 1.03) | 881 | 1.01 (0.98, 1.04) | 181 | 1.00 (0.94, 1.06) |
| T1 (0,0.09) | 356 | 1.0 (reference) | 292 | 1.0 (reference) | 56 | 1.0 (reference) |
| T2 (0.09,0.97) | 353 | 0.98 (0.85, 1.14) | 287 | 0.99 (0.84, 1.16) | 58 | 0.97 (0.67, 1.40) |
| T3 (0.97,26.0) | 374 | 1.04 (0.90, 1.20) | 302 | 1.04 (0.88, 1.22) | 67 | 1.09 (0.76, 1.56) |
| P trend[c] | | 0.74 | | 0.77 | | 0.60 |
| **Bromodichloromethane (µg/L), N=**70734, total person-years=1136722 | | | | | | |
| Log2 | 1083 | 1.02 (1.00, 1.05) | 881 | 1.03 (1.00, 1.05) | 181 | 1.00 (0.95, 1.06) |
| T1 (0,0.23) | 324 | 1.0 (reference) | 264 | 1.0 (reference) | 52 | 1.0 (reference) |
| T2 (0.23,3.34) | 365 | 1.11 (0.96, 1.30) | 292 | 1.12 (0.94, 1.32) | 68 | 1.17 (0.81, 1.69) |
| T3 (3.34,31.0) | 394 | 1.18 (1.01, 1.37) | 325 | 1.22 (1.03, 1.44) | 61 | 1.01 (0.69, 1.46) |
| P trend[c] | | 0.21 | | 0.10 | | 0.53 |
| **Dibromochloromethane (µg/L), N=**70734, total person-years=1136722 | | | | | | |
| Log2 | 1083 | 1.02 (1.00, 1.04) | 881 | 1.02 (1.00, 1.05) | 181 | 1.01 (0.95, 1.06) |
| T1 (0,0.14) | 326 | 1.0 (reference) | 264 | 1.0 (reference) | 55 | 1.0 (reference) |
| T2 (0.14,2.12) | 364 | 1.10 (0.95, 1.28) | 298 | 1.13 (0.96, 1.34) | 59 | 0.96 (0.66, 1.40) |
| T3 (2.12,38.0) | 393 | 1.16 (1.00, 1.35) | 319 | 1.19 (1.01, 1.40) | 67 | 1.05 (0.73, 1.51) |
| P trend[c] | | 0.23 | | 0.22 | | 0.77 |
| **Arsenic (µg/L), N=**69601, total person-years=1117924 | | | | | | |
| Log2 | 1069 | 0.98 (0.94, 1.03) | 869 | 0.99 (0.94, 1.04) | 179 | 0.94 (0.84, 1.05) |
| T1 (0,0.71) | 382 | 1.0 (reference) | 306 | 1.0 (reference) | 69 | 1.0 (reference) |
| T2 (0.71,1.45) | 328 | 0.88 (0.76, 1.02) | 272 | 0.92 (0.78, 1.08) | 50 | 0.73 (0.51, 1.05) |
| T3 (1.45,100) | 359 | 0.94 (0.81, 1.09) | 291 | 0.96 (0.82, 1.13) | 60 | 0.85 (0.60, 1.20) |
| P trend[c] | | 0.90 | | 0.99 | | 0.66 |
| **Uranium (µg/L), N=**63866, total person-years=1022715 | | | | | | |
| Log2 | 977 | 0.98 (0.94, 1.02) | 791 | 0.97 (0.93, 1.02) | 168 | 1.01 (0.91, 1.12) |
| T1 (0.09,3.25) | 349 | 1.0 (reference) | 284 | 1.0 (reference) | 55 | 1.0 (reference) |
| T2 (3.25,5.76) | 330 | 1.00 (0.86, 1.17) | 271 | 1.00 (0.85, 1.18) | 56 | 1.15 (0.79, 1.67) |
| T3 (5.76,101.62) | 298 | 0.88 (0.75, 1.03) | 236 | 0.85 (0.71, 1.01) | 57 | 1.12 (0.77, 1.63) |
| P trend[c] | | 0.16 | | 0.09 | | 0.50 |
| **Nitrate-N (mg/L), N=**69713, total person-years=1119686 | | | | | | |
| Log2 | 1071 | 0.97 (0.94, 1.00) | 870 | 0.97 (0.94, 1.01) | 180 | 0.97 (0.90, 1.04) |

© 2026 Spaur M et al. JAMA Network Open.

| | | | | | | |
|---|---|---|---|---|---|---|
| T1 (0.01,0.27) | 373 | 1.0 (reference) | 300 | 1.0 (reference) | 65 | 1.0 (reference) |
| T2 (0.27,1.40) | 372 | 0.99 (0.86, 1.14) | 304 | 1.01 (0.86, 1.18) | 61 | 0.91 (0.64, 1.30) |
| T3 (1.40,19.22) | 326 | 0.87 (0.75, 1.01) | 266 | 0.88 (0.75, 1.04) | 54 | 0.82 (0.57, 1.18) |
| P trend[c] | | 0.18 | | 0.28 | | 0.47 |

[a]Community water system (CWS) exposures were linked to address(es) from the residential history and represent time-varying cumulative average exposures, lagged 10 years. Participants censored before 2000 were excluded from analyses and follow-up started on Jan 1, 2000.
[b]Model was adjusted for age in 2000 (time varying variable) and age$^2$, BMI category (<25 kg/m$^2$, 25-<30 kg/m$^2$, >30 kg/m$^2$, or missing), and smoking status (never, former, current, or missing).
[c]P trend was evaluated using the median of each tertile.

© 2026 Spaur M et al. JAMA Network Open.

**eTable 12. Hazard ratios (HR, 95% CI) of uterine, endometrioid and non-endometrioid cancers per $log_2$ increase and tertiles of exposures[a], excluding participants censored prior to 2005[b].**

| CWS Concentration Range | N | Uterine Cancer N Cases | Uterine Cancer HR (95% CI)[c] | Endometrioid Tumors N Cases | Endometrioid Tumors HR (95% CI)[c] | Non-Endometrioid Tumors N Cases | Non-Endometrioid Tumors HR (95% CI)[c] |
|---|---|---|---|---|---|---|---|
| **TTHM (µg/L)** | | | | | | | |
| Log2 | 45730 | 635 | 1.04 (1.02, 1.07) | 502 | 1.04 (1.01, 1.07) | 120 | 1.04 (0.98, 1.11) |
| T1 (0,1.22) | 15176 | 177 | 1.0 (reference) | 143 | 1.0 (reference) | 31 | 1.0 (reference) |
| T2 (1.25,15.57) | 15387 | 217 | 1.20 (0.99, 1.47) | 162 | 1.11 (0.89, 1.39) | 49 | 1.54 (0.98, 2.42) |
| T3 (15.83,108.7) | 15167 | 241 | 1.37 (1.13, 1.66) | 197 | 1.39 (1.12, 1.73) | 40 | 1.27 (0.79, 2.03) |
| P trend[d] | | | 0.004 | | 0.002 | | 0.78 |
| **Chloroform (µg/L)** | | | | | | | |
| Log2 | 45730 | 635 | 1.04 (1.02, 1.07) | 502 | 1.05 (1.01, 1.08) | 120 | 1.03 (0.97, 1.10) |
| T1 (0,0.65) | 15226 | 184 | 1.0 (reference) | 144 | 1.0 (reference) | 37 | 1.0 (reference) |
| T2 (0.65,5.77) | 15246 | 213 | 1.14 (0.94, 1.39) | 161 | 1.10 (0.88, 1.38) | 46 | 1.21 (0.79, 1.87) |
| T3 (5.81,82.55) | 15258 | 238 | 1.29 (1.06, 1.57) | 197 | 1.37 (1.11, 1.70) | 37 | 0.97 (0.62, 1.54) |
| P trend[d] | | | 0.01 | | 0.003 | | 0.64 |
| **Bromoform (µg/L)** | | | | | | | |
| Log2 | 45730 | 635 | 1.03 (0.99, 1.07) | 502 | 1.04 (1.00, 1.08) | 120 | 0.99 (0.92, 1.08) |
| T1 (0,0.10) | 15031 | 206 | 1.0 (reference) | 156 | 1.0 (reference) | 44 | 1.0 (reference) |
| T2 (0.10,0.62) | 15256 | 194 | 0.93 (0.77, 1.13) | 157 | 0.99 (0.80, 1.24) | 34 | 0.78 (0.50, 1.21) |
| T3 (0.62,8.78) | 15443 | 235 | 1.13 (0.94, 1.36) | 189 | 1.20 (0.97, 1.48) | 42 | 0.94 (0.62, 1.44) |
| P trend[d] | | | 0.06 | | 0.05 | | 0.79 |
| **Bromodichloromethane (µg/L)** | | | | | | | |
| Log2 | 45730 | 635 | 1.05 (1.02, 1.08) | 502 | 1.06 (1.02, 1.09) | 120 | 1.02 (0.95, 1.09) |
| T1 (0,0.35) | 15262 | 177 | 1.0 (reference) | 134 | 1.0 (reference) | 38 | 1.0 (reference) |
| T2 (0.36,3.23) | 15181 | 224 | 1.29 (1.06, 1.57) | 181 | 1.37 (1.10, 1.72) | 40 | 1.08 (0.69, 1.69) |
| T3 (3.29,20.92) | 15287 | 234 | 1.34 (1.10, 1.62) | 187 | 1.41 (1.13, 1.77) | 42 | 1.10 (0.71, 1.70) |
| P trend[d] | | | 0.03 | | 0.02 | | 0.74 |
| **Dibromochloromethane (µg/L)** | | | | | | | |
| Log2 | 45730 | 635 | 1.03 (1.00, 1.06) | 502 | 1.04 (1.01, 1.08) | 120 | 1.00 (0.94, 1.07) |
| T1 (0,0.20) | 15183 | 188 | 1.0 (reference) | 141 | 1.0 (reference) | 41 | 1.0 (reference) |
| T2 (0.20,1.69) | 15191 | 211 | 1.12 (0.92, 1.37) | 172 | 1.22 (0.98, 1.53) | 37 | 0.90 (0.58, 1.41) |
| T3 (1.70,17.85) | 15356 | 236 | 1.26 (1.04, 1.52) | 189 | 1.34 (1.08, 1.67) | 42 | 1.01 (0.66, 1.56) |
| P trend[d] | | | 0.03 | | 0.03 | | 0.76 |
| **Arsenic (µg/L)** | | | | | | | |
| Log2 | 45692 | 634 | 0.98 (0.92, 1.05) | 501 | 0.99 (0.92, 1.06) | 120 | 0.95 (0.81, 1.11) |
| T1 (0.01,0.70) | 15088 | 221 | 1.0 (reference) | 169 | 1.0 (reference) | 47 | 1.0 (reference) |
| T2 (0.70,1.61) | 14470 | 189 | 0.89 (0.74, 1.09) | 150 | 0.93 (0.74, 1.15) | 36 | 0.81 (0.53, 1.25) |
| T3 (1.62,36.55) | 16134 | 224 | 0.94 (0.78, 1.13) | 182 | 1.00 (0.81, 1.23) | 37 | 0.74 (0.48, 1.14) |
| P trend[d] | | | 0.70 | | 0.86 | | 0.21 |
| **Uranium (µg/L)** | | | | | | | |
| Log2 | 42921 | 594 | 1.01 (0.96, 1.06) | 467 | 1.01 (0.95, 1.07) | 116 | 1.01 (0.90, 1.14) |
| T1 (0.09,2.63) | 14226 | 195 | 1.0 (reference) | 151 | 1.0 (reference) | 38 | 1.0 (reference) |
| T2 (2.64,5.18) | 14401 | 202 | 1.05 (0.87, 1.28) | 165 | 1.11 (0.89, 1.38) | 36 | 0.99 (0.62, 1.56) |
| T3 (5.20,100.93) | 14294 | 197 | 1.02 (0.83, 1.24) | 151 | 1.01 (0.80, 1.26) | 42 | 1.12 (0.72, 1.74) |
| P trend[d] | | | 0.94 | | 0.89 | | 0.56 |

© 2026 Spaur M et al. JAMA Network Open.

| | | | | | | | |
|---|---|---|---|---|---|---|---|
| **Nitrate-N (mg/L)** | | | | | | | |
| Log2 | 45704 | 634 | 0.97 (0.93, 1.01) | 501 | 0.97 (0.93, 1.02) | 120 | 0.94 (0.86, 1.03) |
| T1 (0.01,0.25) | 15031 | 215 | 1.0 (reference) | 164 | 1.0 (reference) | 48 | 1.0 (reference) |
| T2 (0.25,1.53) | 15394 | 224 | 0.99 (0.82, 1.19) | 187 | 1.08 (0.87, 1.33) | 31 | 0.62 (0.40, 0.98) |
| T3 (1.53,9.62) | 15279 | 195 | 0.86 (0.71, 1.05) | 150 | 0.87 (0.70, 1.08) | 41 | 0.83 (0.55, 1.26) |
| P trend[d] | | | 0.11 | | 0.09 | | 0.87 |

[a]Community water system (CWS) exposures were linked to the address at enrollment and represent 1990-2005 average concentrations of individual and total trihalomethanes (TTHM), arsenic, uranium, and nitrate-N.
[b]Analyses were restricted to participants with a residential duration at enrollment ≥ 10 years. Participants who were censored prior to 2005 were excluded. Person time was re-calculated so that follow-up time started on Jan 1, 2005, and age was re-calculated to reflect age on Jan 1, 2005 (baseline age + years between the participant start date and Jan 1, 2005). Total person-years = 584074.6; mean follow-up=12.8 years.
[c]Model was adjusted for age (years) and age$^2$, and smoking status (never, former, current, or missing). P trend was evaluated using the median of each quantile.
[d]P trend was evaluated using the median of each tertile.

© 2026 Spaur M et al. JAMA Network Open.

**eTable 13. Hazard ratios (HR, 95% CI) of uterine, endometrioid, and non-endometrioid cancers per $\log_2$ increase and tertiles of average (1990-2013) trihalomethane exposures[a,b].**

| | | Uterine Cancer | | Endometrioid Tumors | | Non-Endometrioid Tumors | |
|---|---|---|---|---|---|---|---|
| **Conc. Range (µg/L)** | **N** | **N Cases** | **HR (95% CI)[b]** | **N Cases** | **HR (95% CI)[b]** | **N Cases** | **HR (95% CI)[b]** |
| **TTHM** | | | | | | | |
| Log2 | 39900 | 412 | 1.05 (1.00, 1.10) | 312 | 1.04 (0.99, 1.10) | 91 | 1.09 (0.99, 1.21) |
| T1 (0,5.35) | 13291 | 116 | 1.0 (reference) | 94 | 1.0 (reference) | 19 | 1.0 (reference) |
| T2 (5.35,17.0) | 10989 | 109 | 1.15 (0.89, 1.50) | 74 | 0.97 (0.71, 1.31) | 32 | 2.06 (1.17, 3.63) |
| T3 (17.15,98.83) | 15620 | 187 | 1.41 (1.12, 1.78) | 144 | 1.34 (1.04, 1.74) | 40 | 1.81 (1.05, 3.12) |
| P trend[d] | | | 0.003 | | 0.01 | | 0.08 |
| **Chloroform** | | | | | | | |
| Log2 | 39900 | 412 | 1.05 (1.01, 1.10) | 312 | 1.04 (0.99, 1.09) | 91 | 1.10 (1.01, 1.20) |
| T1 (0,1.66) | 13325 | 115 | 1.0 (reference) | 91 | 1.0 (reference) | 23 | 1.0 (reference) |
| T2 (1.68,6.76) | 13203 | 138 | 1.23 (0.96, 1.58) | 109 | 1.24 (0.93, 1.63) | 26 | 1.15 (0.66, 2.02) |
| T3 (6.82,85.28) | 13372 | 159 | 1.43 (1.12, 1.82) | 112 | 1.28 (0.97, 1.69) | 42 | 1.84 (1.11, 3.07) |
| P trend[d] | | | 0.01 | | 0.13 | | 0.01 |
| **Bromoform** | | | | | | | |
| Log2 | 39900 | 412 | 1.02 (0.98, 1.07) | 312 | 1.04 (0.99, 1.10) | 91 | 0.97 (0.88, 1.07) |
| T1 (0,0.20) | 12921 | 132 | 1.0 (reference) | 95 | 1.0 (reference) | 32 | 1.0 (reference) |
| T2 (0.20,1.54) | 13642 | 136 | 0.98 (0.77, 1.25) | 106 | 1.06 (0.80, 1.40) | 28 | 0.84 (0.51, 1.40) |
| T3 (1.55,13.89) | 13337 | 144 | 1.07 (0.85, 1.36) | 111 | 1.14 (0.87, 1.51) | 31 | 0.96 (0.58, 1.57) |
| P trend[d] | | | 0.48 | | 0.35 | | 0.95 |
| **Bromodichloromethane** | | | | | | | |
| Log2 | 39900 | 412 | 1.06 (1.01, 1.11) | 312 | 1.06 (1.01, 1.11) | 91 | 1.06 (0.97, 1.17) |
| T1 (0,0.72) | 12813 | 110 | 1.0 (reference) | 81 | 1.0 (reference) | 25 | 1.0 (reference) |
| T2 (0.72,4.70) | 13693 | 148 | 1.28 (1.00, 1.64) | 113 | 1.32 (1.00, 1.76) | 32 | 1.22 (0.72, 2.06) |
| T3 (4.72,20.32) | 13394 | 154 | 1.38 (1.08, 1.77) | 118 | 1.44 (1.09, 1.92) | 34 | 1.32 (0.79, 2.21) |
| P trend[d] | | | 0.03 | | 0.03 | | 0.35 |
| **Dibromochloromethane** | | | | | | | |
| Log2 | 39900 | 412 | 1.03 (0.99, 1.07) | 312 | 1.04 (1.00, 1.09) | 91 | 1.02 (0.93, 1.11) |
| T1 (0,0.57) | 13325 | 121 | 1.0 (reference) | 88 | 1.0 (reference) | 28 | 1.0 (reference) |
| T2 (0.57,3.19) | 13198 | 142 | 1.22 (0.96, 1.55) | 106 | 1.25 (0.94, 1.66) | 34 | 1.26 (0.76, 2.08) |
| T3 (3.22,18.82) | 13377 | 149 | 1.26 (0.99, 1.60) | 118 | 1.37 (1.04, 1.81) | 29 | 1.05 (0.63, 1.77) |
| P trend[d] | | | 0.14 | | 0.05 | | 0.88 |

[a]Community water system (CWS) exposures were linked to the address at enrollment and represent 1990-2013 average concentrations (µg/L) of individual and total trihalomethanes (TTHM).

© 2026 Spaur M et al. JAMA Network Open.

[b]Analyses were restricted to participants with a residential duration at enrollment ≥ 10 years. Participants who were censored prior to 2010 were excluded. Person time was re-calculated so that follow-up time started on Jan 1, 2010, and age was re-calculated to reflect age on Jan 1, 2010 (baseline age + years between the participant start date and Jan 1, 2010). Total person-years = 370167.5; mean follow-up=9.3 years.
[c]Model was adjusted for age in 2010 (years) and age$^2$, BMI category (<25 kg/m$^2$, 25-<30 kg/m$^2$, >30 kg/m$^2$, or missing), and smoking status (never, former, current, or missing).
[d]P trend was evaluated using the median of each tertile.

© 2026 Spaur M et al. JAMA Network Open.

**eTable 14. Hazard Ratios (HR, 95% CIs) of uterine, endometrioid and non-endometrioid cancers per $\log_2$ increase in exposures[a] among participants with exposures less than maximum contaminant level (MCL)[b].**

| | | Uterine Cancer | | Endometrioid Tumors | | Non-Endometrioid Tumors | |
|---|---|---|---|---|---|---|---|
| | N | N cases | HR (95% CI)[c] | N Cases | HR (95% CI)[c] | N cases | HR (95% CI)[c] |
| TTHM | 53065 | 1038 | 1.02 (1.00, 1.04) | 864 | 1.03 (1.00, 1.05) | 154 | 1.00 (0.95, 1.05) |
| Chloroform | 53065 | 1038 | 1.03 (1.01, 1.05) | 864 | 1.04 (1.01, 1.06) | 154 | 0.99 (0.93, 1.05) |
| Bromoform | 53065 | 1038 | 1.01 (0.98, 1.04) | 864 | 1.02 (0.99, 1.05) | 154 | 0.97 (0.90, 1.04) |
| Bromodichloromethane | 53065 | 1038 | 1.02 (1.00, 1.05) | 864 | 1.03 (1.01, 1.06) | 154 | 0.98 (0.93, 1.05) |
| Dibromochloromethane | 53065 | 1038 | 1.01 (0.99, 1.04) | 864 | 1.02 (1.00, 1.05) | 154 | 0.98 (0.92, 1.04) |
| Arsenic | 52606 | 1028 | 0.98 (0.93, 1.04) | 857 | 0.98 (0.92, 1.04) | 152 | 0.98 (0.85, 1.13) |
| Uranium | 49786 | 973 | 0.98 (0.94, 1.02) | 806 | 0.97 (0.93, 1.01) | 149 | 1.03 (0.92, 1.14) |
| Nitrate-N | 53073 | 1037 | 0.99 (0.96, 1.02) | 863 | 0.99 (0.96, 1.02) | 154 | 0.97 (0.90, 1.05) |
| **Mixture[d]** | | | | | | | |
| Chloroform + Bromoform + Brdichl + Dichlbr + Arsenic + Uranium + Nitrate-N | 49394 | 964 | 1.24 (0.98, 1.57) | 800 | 1.37 (1.06, 1.77) | 147 | 0.68 (0.36, 1.28) |
| TTHM + Arsenic + Uranium + Nitrate-N | 49394 | 964 | 1.17 (0.94, 1.45) | 800 | 1.21 (0.95, 1.54) | 147 | 0.89 (0.51, 1.55) |

[a]Community water system (CWS) exposures were linked to the address at enrollment and represent 1990-2005 average concentrations of individual and total trihalomethanes (TTHM, µg/L), arsenic (µg/L), uranium (µg/L), and nitrate-N (mg/L). Analyses were restricted to participants with a residential duration at enrollment ≥ 10 years.

[b]Analyses were further restricted to participants with 1990-2005 average nitrate, arsenic, uranium, or TTHM concentrations less than the corresponding MCLs: 80 µg/L (TTHM), 30 µg/L (uranium), 10 µg/L (arsenic), and 10 mg/L (nitrate).

[c]Model was adjusted for baseline age (years) and age$^2$, BMI category (<25 kg/m2, 25-<30 kg/m2, >30 kg/m2, or missing), and smoking status (never, former, current, or missing).

[d]In the mixtures models, all contaminant averages were < MCLs. Concentrations were log2 transformed and subsequently divided by the IQR. The effect of the drinking water contaminant mixture was evaluated using quantile q-computation, and hazards ratios are interpreted per IQR increase.

© 2026 Spaur M et al. JAMA Network Open.

**eTable 15. Hazard Ratios (HR, 95% CIs) of uterine, endometrioid and non-endometrioid cancers per $log_2$ increase and tertiles of exposures using mixed-effects models[a,b]**

| | | Uterine Cancer | | Endometrioid Tumors | | Non-Endometrioid Tumors | |
|---|---|---|---|---|---|---|---|
| | N | N Cases | HR (95% CI)[c] | N Cases | HR (95% CI)[c] | N Cases | HR (95% CI)[c] |
| **TTHM** | | | | | | | |
| Log2 | 53100 | 1038 | 1.02 (1.00, 1.04) | 864 | 1.02 (1.00, 1.05) | 154 | 1.00 (0.95, 1.06) |
| T1 (0,1.22) | 17571 | 307 | 1.0 (reference) | 253 | 1.0 (reference) | 48 | 1.0 (reference) |
| T2 (1.25,15.57) | 17825 | 355 | 1.12 (0.95, 1.31) | 287 | 1.09 (0.92, 1.29) | 59 | 1.19 (0.80, 1.75) |
| T3 (15.83,108.66) | 17704 | 376 | 1.21 (1.08, 1.36) | 324 | 1.24 (1.09, 1.40) | 47 | 0.96 (0.72, 1.27) |
| P trend[d] | | | 0.04 | | 0.01 | | 0.59 |
| **Chloroform** | | | | | | | |
| Log2 | 53100 | 1038 | 1.03 (1.01, 1.05) | 864 | 1.04 (1.01, 1.06) | 154 | 0.99 (0.93, 1.05) |
| T1 (0,0.65) | 17570 | 303 | 1.0 (reference) | 245 | 1.0 (reference) | 52 | 1.0 (reference) |
| T2 (0.65,5.77) | 17728 | 360 | 1.15 (0.98, 1.34) | 294 | 1.15 (0.97, 1.37) | 57 | 1.07 (0.73, 1.57) |
| T3 (5.81,82.55) | 17802 | 375 | 1.20 (1.07, 1.35) | 325 | 1.27 (1.12, 1.44) | 45 | 0.83 (0.63, 1.10) |
| P trend[d] | | | 0.06 | | 0.01 | | 0.29 |
| **Bromoform** | | | | | | | |
| Log2 | 53100 | 1038 | 1.01 (0.98, 1.04) | 864 | 1.02 (0.99, 1.05) | 154 | 0.98 (0.90, 1.05) |
| T1 (0,0.10) | 17586 | 350 | 1.0 (reference) | 285 | 1.0 (reference) | 57 | 1.0 (reference) |
| T2 (0.10,0.62) | 17620 | 331 | 0.95 (0.81, 1.12) | 276 | 0.98 (0.83, 1.16) | 47 | 0.83 (0.56, 1.25) |
| T3 (0.62,8.78) | 17894 | 357 | 1.02 (0.91, 1.14) | 303 | 1.05 (0.93, 1.19) | 50 | 0.89 (0.67, 1.18) |
| P trend[d] | | | 0.55 | | 0.40 | | 0.88 |
| **Bromodichloromethane** | | | | | | | |
| Log2 | 53100 | 1038 | 1.03 (1.00, 1.05) | 864 | 1.03 (1.01, 1.06) | 154 | 0.99 (0.92, 1.05) |
| T1 (0,0.35) | 17695 | 314 | 1.0 (reference) | 250 | 1.0 (reference) | 54 | 1.0 (reference) |
| T2 (0.36,3.23) | 17592 | 357 | 1.15 (0.99, 1.34) | 301 | 1.22 (1.03, 1.44) | 51 | 0.95 (0.64, 1.41) |
| T3 (3.29,20.92) | 17813 | 367 | 1.14 (1.02, 1.27) | 313 | 1.22 (1.08, 1.38) | 49 | 0.89 (0.68, 1.18) |
| P trend[d] | | | 0.25 | | 0.11 | | 0.62 |
| **Dibromochloromethane** | | | | | | | |
| Log2 | 53100 | 1038 | 1.02 (0.99, 1.04) | 864 | 1.02 (1.00, 1.05) | 154 | 0.98 (0.92, 1.05) |
| T1 (0,0.20) | 17638 | 323 | 1.0 (reference) | 256 | 1.0 (reference) | 57 | 1.0 (reference) |
| T2 (0.20,1.69) | 17661 | 357 | 1.08 (0.93, 1.26) | 304 | 1.17 (0.99, 1.38) | 48 | 0.81 (0.54, 1.21) |
| T3 (1.70,17.85) | 17801 | 358 | 1.09 (0.97, 1.21) | 304 | 1.16 (1.02, 1.31) | 49 | 0.86 (0.66, 1.13) |
| P trend[d] | | | 0.44 | | 0.30 | | 0.71 |
| **Arsenic (μg/L)** | | | | | | | |
| Log2 | 53060 | 1037 | 0.99 (0.93, 1.04) | 863 | 0.98 (0.92, 1.03) | 154 | 1.00 (0.86, 1.15) |
| T1 (0.01,0.70) | 17507 | 365 | 1.0 (reference) | 304 | 1.0 (reference) | 54 | 1.0 (reference) |
| T2 (0.70,1.61) | 16760 | 306 | 0.90 (0.77, 1.05) | 256 | 0.91 (0.77, 1.07) | 47 | 0.93 (0.61, 1.41) |
| T3 (1.62,36.55) | 18793 | 366 | 0.95 (0.86, 1.06) | 303 | 0.95 (0.85, 1.06) | 53 | 0.92 (0.69, 1.24) |
| P trend[d] | | | 0.78 | | 0.72 | | 0.74 |
| **Uranium (μg/L)** | | | | | | | |
| Log2 | 49831 | 974 | 0.98 (0.94, 1.02) | 807 | 0.97 (0.93, 1.01) | 149 | 1.04 (0.92, 1.16) |
| T1 (0.09,2.63) | 16592 | 343 | 1.0 (reference) | 288 | 1.0 (reference) | 47 | 1.0 (reference) |
| T2 (2.64,5.18) | 16600 | 317 | 0.98 (0.84, 1.15) | 264 | 0.97 (0.82, 1.15) | 49 | 1.13 (0.73, 1.73) |
| T3 (5.20,100.93) | 16639 | 314 | 0.94 (0.84, 1.05) | 255 | 0.91 (0.81, 1.02) | 53 | 1.15 (0.84, 1.58) |

© 2026 Spaur M et al. JAMA Network Open.

| | | | | | | | |
|---|---|---|---|---|---|---|---|
| P trend[d] | | | 0.41 | | 0.24 | | 0.56 |
| **Nitrate-N (mg/L)** | | | | | | | |
| Log2 | 53073 | 1037 | 0.99 (0.96, 1.02) | 863 | 0.99 (0.96, 1.02) | 154 | 0.97 (0.90, 1.06) |
| T1 (0.01,0.25) | 17539 | 351 | 1.0 (reference) | 289 | 1.0 (reference) | 57 | 1.0 (reference) |
| T2 (0.25,1.53) | 17814 | 352 | 0.98 (0.84, 1.15) | 299 | 1.02 (0.86, 1.19) | 45 | 0.77 (0.51, 1.17) |
| T3 (1.53,9.62) | 17720 | 334 | 0.95 (0.85, 1.06) | 275 | 0.95 (0.84, 1.06) | 52 | 0.89 (0.67, 1.19) |
| P trend[d] | | | 0.49 | | 0.43 | | 0.93 |
| **HAA5 (µg/L)** | | | | | | | |
| Log2 | 33240 | 331 | 1.03 (0.96, 1.10) | 253 | 0.98 (0.91, 1.06) | 72 | 1.19 (1.01, 1.39) |
| T1 (0.35,4.60) | 11098 | 104 | 1.0 (reference) | 84 | 1.0 (reference) | 19 | 1.0 (reference) |
| T2 (4.64,13.10) | 10965 | 106 | 1.07 (0.81, 1.4) | 87 | 1.08 (0.80, 1.46) | 18 | 1.00 (0.52, 1.91) |
| T3 (13.20,115.18) | 11177 | 121 | 1.18 (0.98, 1.43) | 82 | 0.99 (0.80, 1.23) | 35 | 1.86 (1.19, 2.92) |
| P trend[d] | | | 0.21 | | 0.9 | | 0.01 |
| **Monobromoacetic acid (µg/L)** | | | | | | | |
| Log2 | 33280 | 330 | 1.19 (1.05, 1.34) | 252 | 1.21 (1.05, 1.38) | 72 | 1.05 (0.80, 1.38) |
| T1 (0.02,0.25) | 11119 | 107 | 1.0 (reference) | 79 | 1.0 (reference) | 27 | 1.0 (reference) |
| T2 (0.25,0.30) | 10571 | 98 | 0.98 (0.75, 1.29) | 76 | 1.03 (0.75, 1.41) | 21 | 0.84 (0.48, 1.49) |
| T3 (0.30,4.60) | 11590 | 125 | 1.13 (0.94, 1.37) | 97 | 1.19 (0.95, 1.48) | 24 | 0.87 (0.60, 1.27) |
| P trend[d] | | | 0.29 | | 0.23 | | 0.67 |
| **Monochloroacetic acid (µg/L)** | | | | | | | |
| Log2 | 33303 | 331 | 1.04 (0.98, 1.11) | 253 | 1.01 (0.94, 1.08) | 72 | 1.14 (1.00, 1.29) |
| T1 (0,0.10) | 9931 | 101 | 1.0 (reference) | 85 | 1.0 (reference) | 15 | 1.0 (reference) |
| T2 (0.10,0.26) | 11940 | 108 | 0.88 (0.67, 1.16) | 82 | 0.79 (0.59, 1.08) | 25 | 1.38 (0.73, 2.62) |
| T3 (0.27,6.78) | 11432 | 122 | 1.05 (0.87, 1.28) | 86 | 0.88 (0.71, 1.09) | 32 | 1.86 (1.12, 3.09) |
| P trend[d] | | | 0.36 | | 0.81 | | 0.05 |
| **Dibromoacetic acid (µg/L)** | | | | | | | |
| Log2 | 33303 | 331 | 1.02 (0.95, 1.10) | 253 | 1.02 (0.93, 1.10) | 72 | 1.03 (0.88, 1.20) |
| T1 (0.03,0.73) | 11054 | 106 | 1.0 (reference) | 80 | 1.0 (reference) | 24 | 1.0 (reference) |
| T2 (0.75,2.55) | 10468 | 105 | 1.04 (0.79, 1.36) | 78 | 1.02 (0.74, 1.39) | 26 | 1.16 (0.66, 2.02) |
| T3 (2.56,30.0) | 11781 | 120 | 1.07 (0.89, 1.30) | 95 | 1.12 (0.90, 1.40) | 22 | 0.88 (0.59, 1.31) |
| P trend[d] | | | 0.6 | | 0.41 | | 0.57 |
| **Dichloroacetic acid (µg/L)** | | | | | | | |
| Log2 | 33303 | 331 | 1.02 (0.96, 1.09) | 253 | 0.99 (0.91, 1.06) | 72 | 1.16 (0.99, 1.34) |
| T1 (0.03,1.82) | 11016 | 105 | 1.0 (reference) | 85 | 1.0 (reference) | 18 | 1.0 (reference) |
| T2 (1.84,5.60) | 11030 | 115 | 1.11 (0.86, 1.45) | 91 | 1.09 (0.81, 1.46) | 23 | 1.31 (0.71, 2.43) |
| T3 (5.71,38.41) | 11257 | 111 | 1.06 (0.87, 1.28) | 77 | 0.91 (0.73, 1.12) | 31 | 1.71 (1.08, 2.71) |
| P trend[d] | | | 0.75 | | 0.47 | | 0.07 |
| **Trichloroacetic acid (µg/L)** | | | | | | | |
| Log2 | 33305 | 331 | 1.04 (0.98, 1.11) | 253 | 1.02 (0.95, 1.09) | 72 | 1.11 (0.97, 1.28) |

© 2026 Spaur M et al. JAMA Network Open.

| | | | | | | | |
|---|---|---|---|---|---|---|---|
| T1 (0.04,1.32) | 11113 | 102 | 1.0 (reference) | 83 | 1.0 (reference) | 18 | 1.0 (reference) |
| T2 (1.35,4.34) | 11013 | 118 | 1.19 (0.91, 1.55) | 90 | 1.12 (0.83, 1.50) | 27 | 1.54 (0.85, 2.80) |
| T3 (4.37,87.78) | 11179 | 111 | 1.11 (0.91, 1.35) | 80 | 0.98 (0.79, 1.22) | 27 | 1.51 (0.95, 2.39) |
| P trend[d] | | | 0.58 | | 0.82 | | 0.25 |

[a]Community water system exposures were linked to the address at enrollment and represent 1990-2005 average concentrations of trihalomethanes and inorganics, and 1990-2013 concentrations of haloacetic acids.
[b]Analyses were restricted to participants with a residential duration at enrollment $\geq$ 10 years. Haloacetic acid analyses started follow-up in 2010.
[c]Model was adjusted for baseline age (years) and age$^2$, BMI category (<25 kg/m$^2$, 25-<30 kg/m$^2$, >30 kg/m$^2$, or missing), and smoking status (never, former, current, or missing), and included a random effect for the CWS identifier.
[d]P trend was evaluated using the median of each tertile.

© 2026 Spaur M et al. JAMA Network Open.

**eFigure 1. Exclusion and inclusion criteria.**

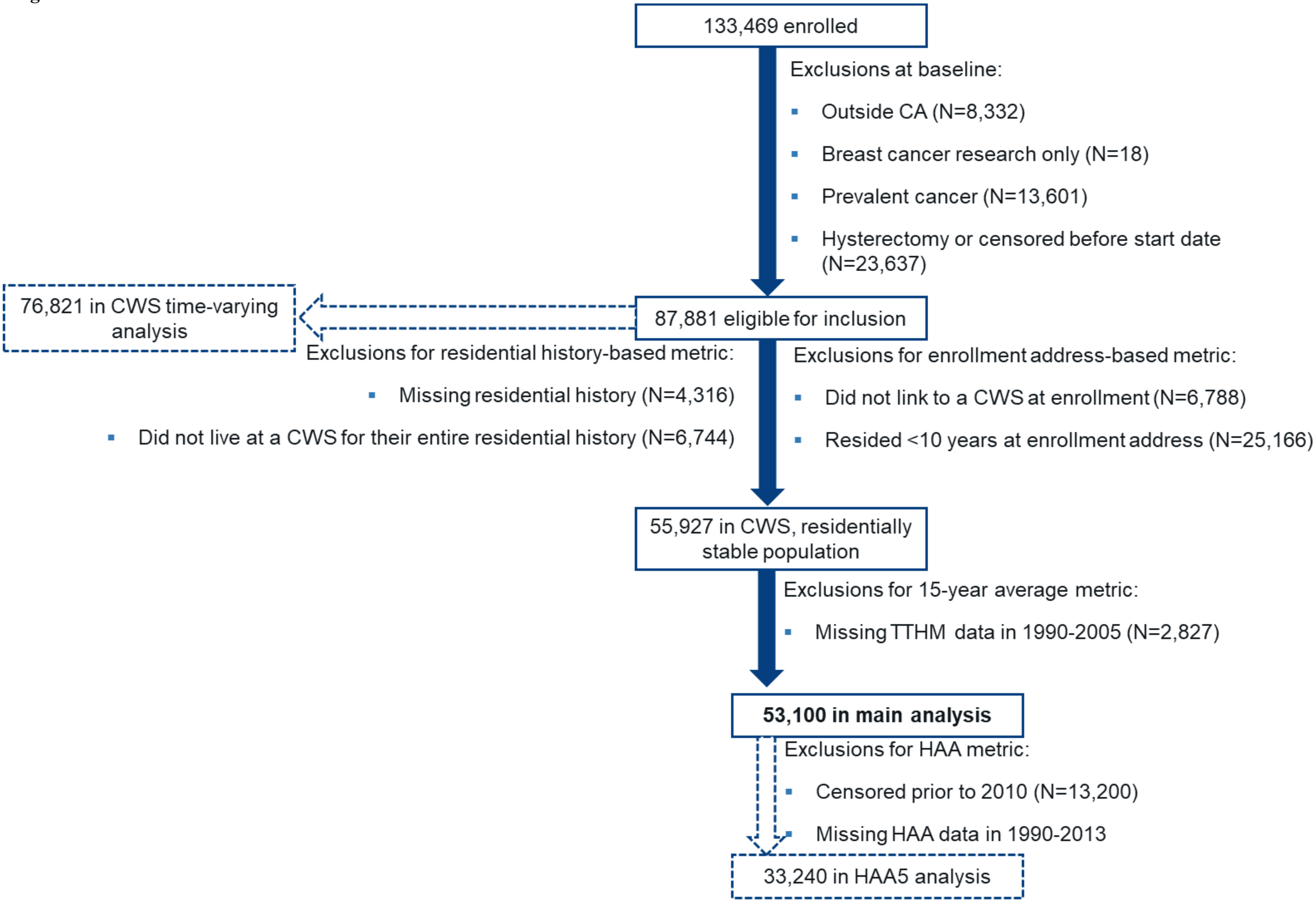


© 2026 Spaur M et al. JAMA Network Open.

**eFigure 2. Number (N) of community water systems (CWS) reporting annual water quality data by year and contaminant, linked to the enrollment address.**

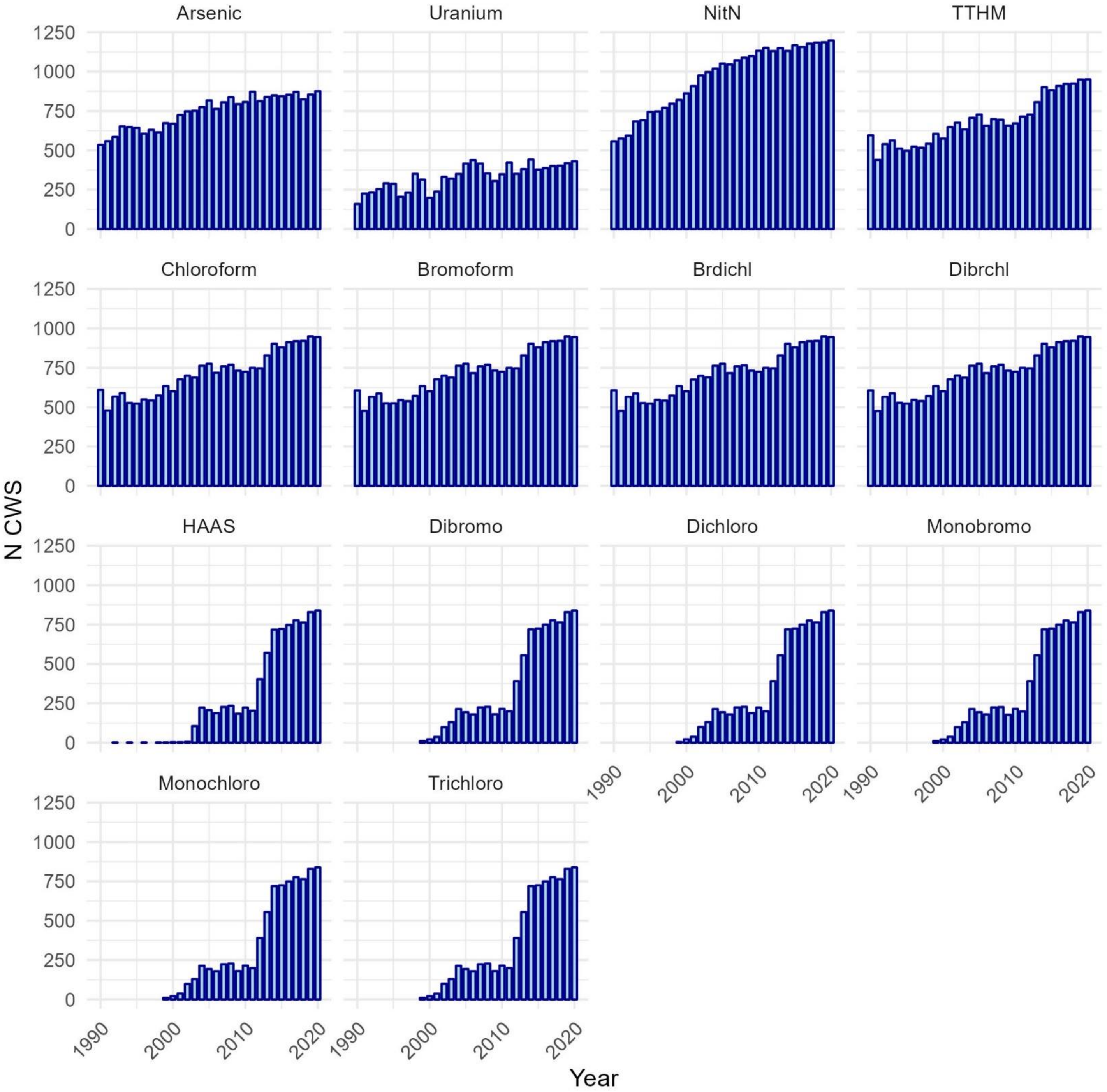


Contaminants are arsenic, uranium, nitrate-nitrogen (NitN), individual trihalomethanes (chloroform, bromoform, dibromochloromethane [Dibrchl], and bromodichloromethane [Brdichl]), and total trihalomethanes (TTHM), individual haloacetic acids (monochloroacetic acid [Monochloro], dichloroacetic acid [Dichloro], trichloroacetic acid [Trichloro], monobromoacetic acid [Monobromo], and dibromoacetic acid [Dibromo]) and the sum of five (HAAS).

© 2026 Spaur M et al. JAMA Network Open.

**eFigure 3. Directed acyclic graph (DAG) of exposures, outcomes, and measured and unmeasured confounders.**

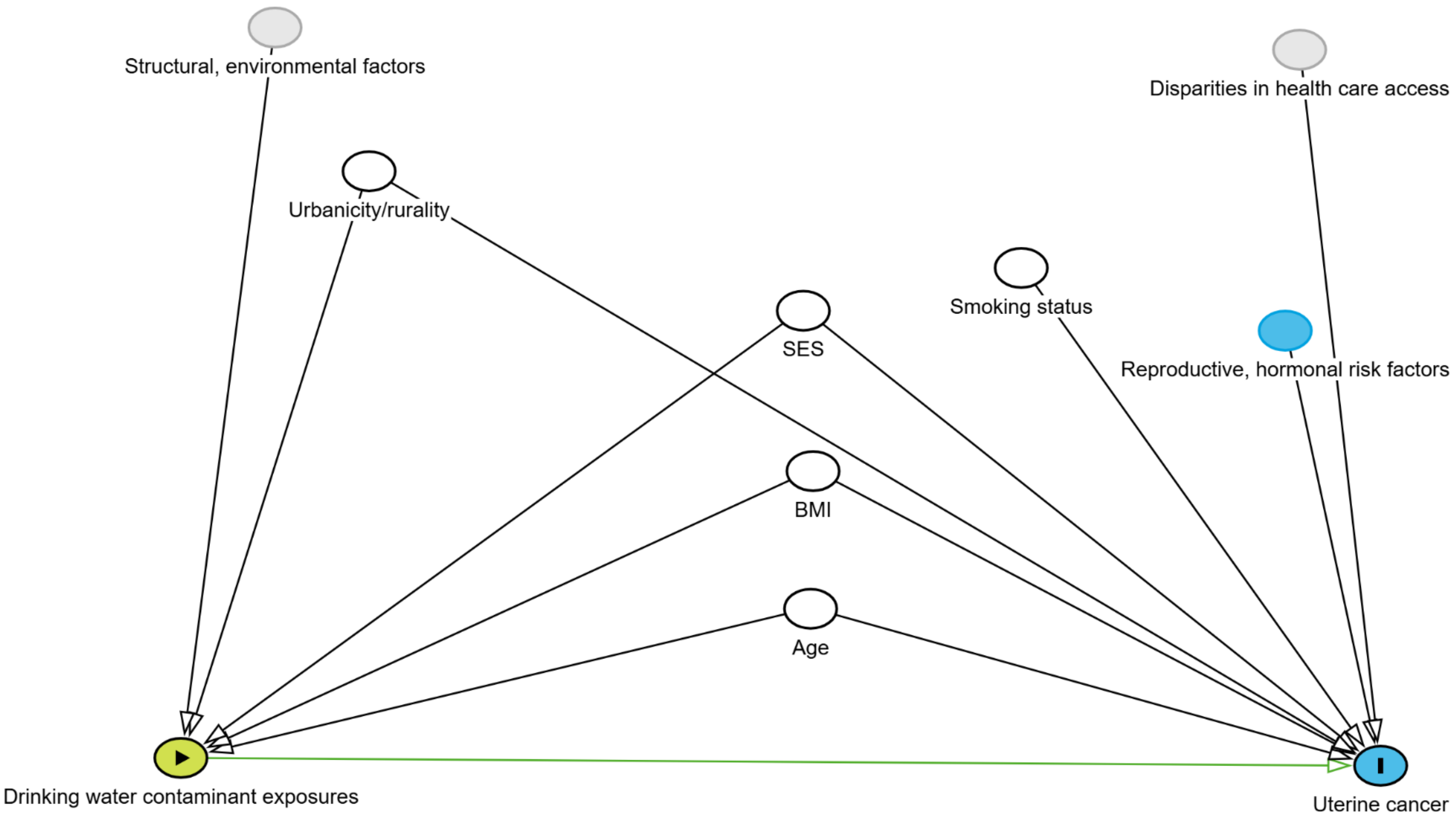


White ovals represent confounding factors; gray ovals represent latent (unobserved) variables; the green oval represents the exposure; the blue oval represents the outcome and other outcome-specific risk factors.

© 2026 Spaur M et al. JAMA Network Open.

**eFigure 4. Spearman correlation coefficients of 15-year average (1990-2005) concentrations of community water system arsenic, uranium, nitrate-N (NitrateN), individual trihalomethanes [chloroform, bromoform, dibromochloromethane [Dibrchl], Bromodichloromethane [Brdichl]) and total trihalomethanes (TTHM), and 1990-2013 average concentrations of individual haloacetic acids [monobromo-, monochloro-, dibromo-, dichloro-, trichloro], and their sum (HAA5).**

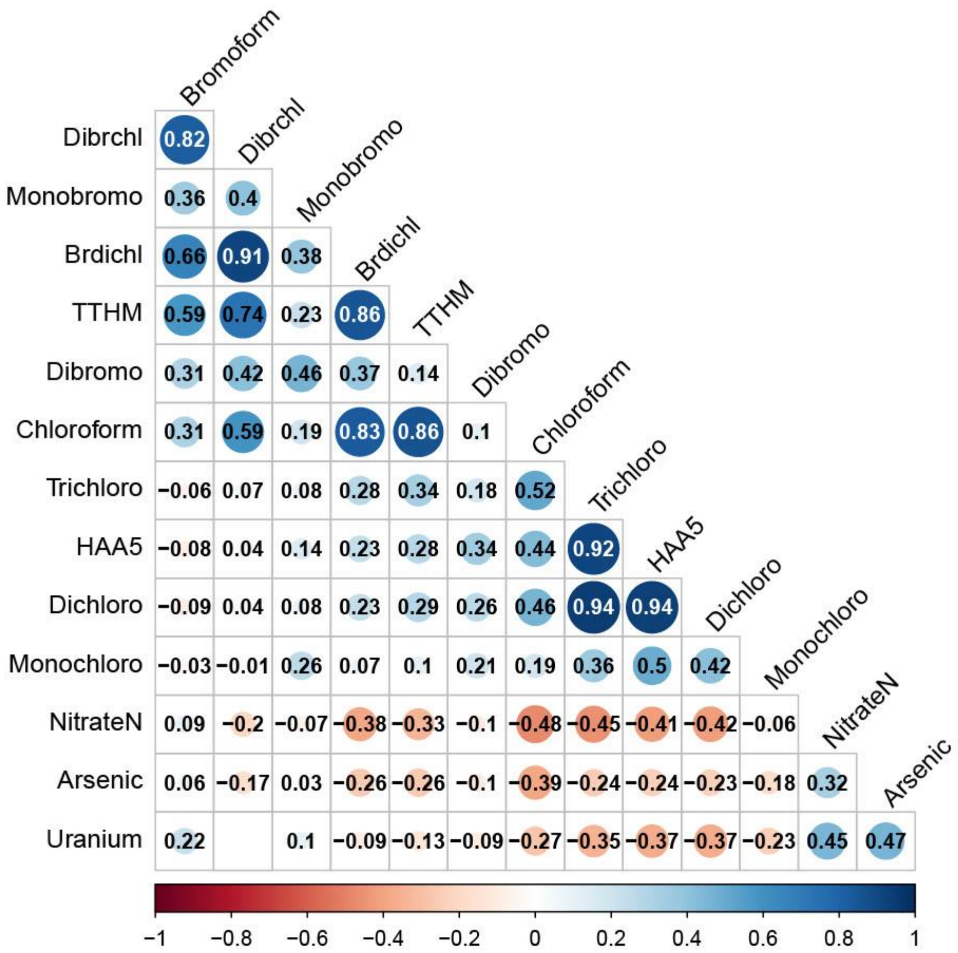


Positive correlations are shown in blue and negative correlations are shown in red. Blank cells indicate no statistically significant correlation (p>0.05).

© 2026 Spaur M et al. JAMA Network Open.

**eFigure 5. Exposure-response associations between community water system (CWS) nitrate, uranium, arsenic, total trihalomethanes (TTHM), and individual trihalomethanes (chloroform, bromoform, dibromochloromethane [Dibrchl], and bromodichloromethane [Brdichl]) and uterine cancer.**

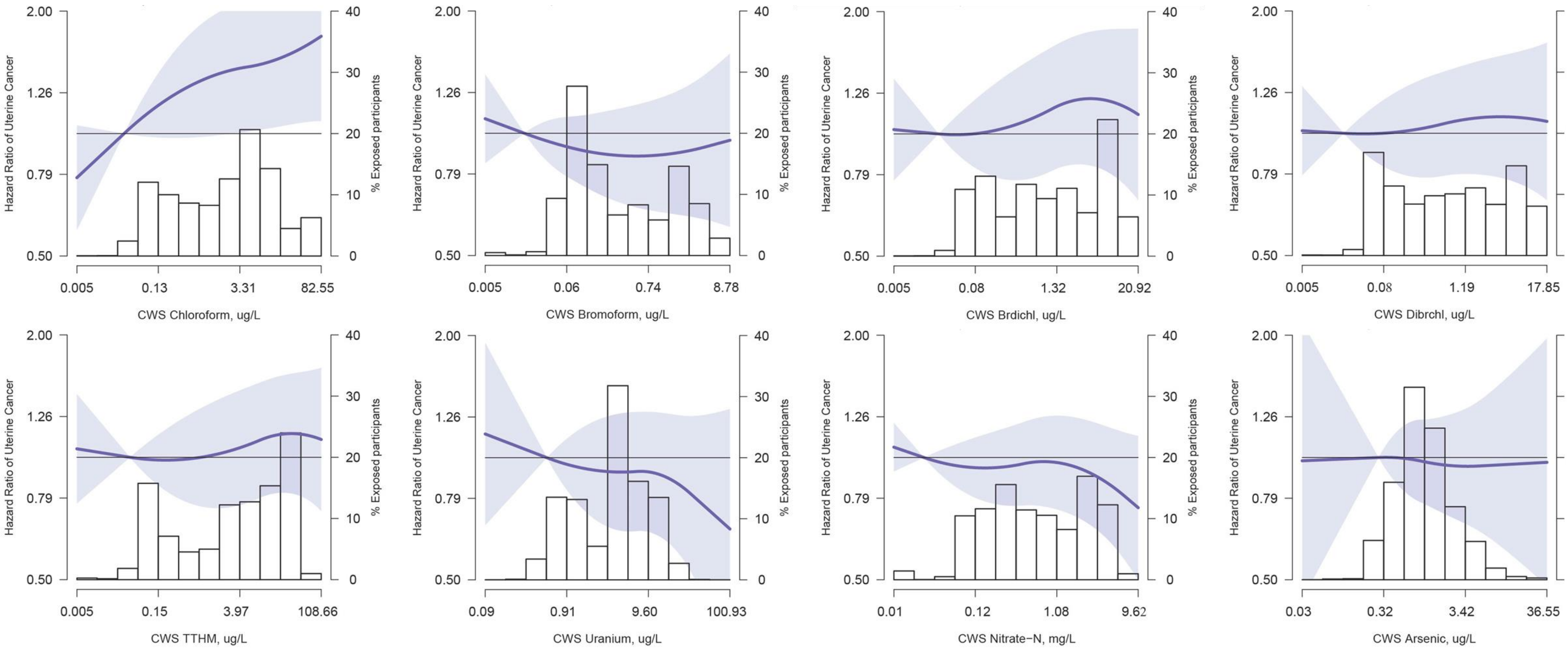


Lines with shaded areas represent the hazard ratios (HR) and 95% confidence intervals, based on cubic splines for Cox proportional hazards models using log2-transformed concentrations with knots at the 10th (reference), 50th, and 90th percentiles. A black vertical line is at HR=1. The histogram represents the frequency distribution of exposures in the study sample. CWS exposures were linked to the address at enrollment. Analyses were restricted to participants with a residential duration at enrollment ≥ 10 years. Model was adjusted for baseline age (years) and baseline age$^2$, BMI category (<25 kg/m$^2$, 25-<30 kg/m$^2$, >30 kg/m$^2$, or missing), and smoking status (never, former, current, or missing).

© 2026 Spaur M et al. JAMA Network Open.

**eFigure 6. Exposure-response associations between community water system (CWS) nitrate, uranium, arsenic, total trihalomethanes (TTHM), and individual trihalomethanes (chloroform, bromoform, dibromochloromethane [Dibrchl], and bromodichloromethane [Brdichl]) and endometrioid tumors.**

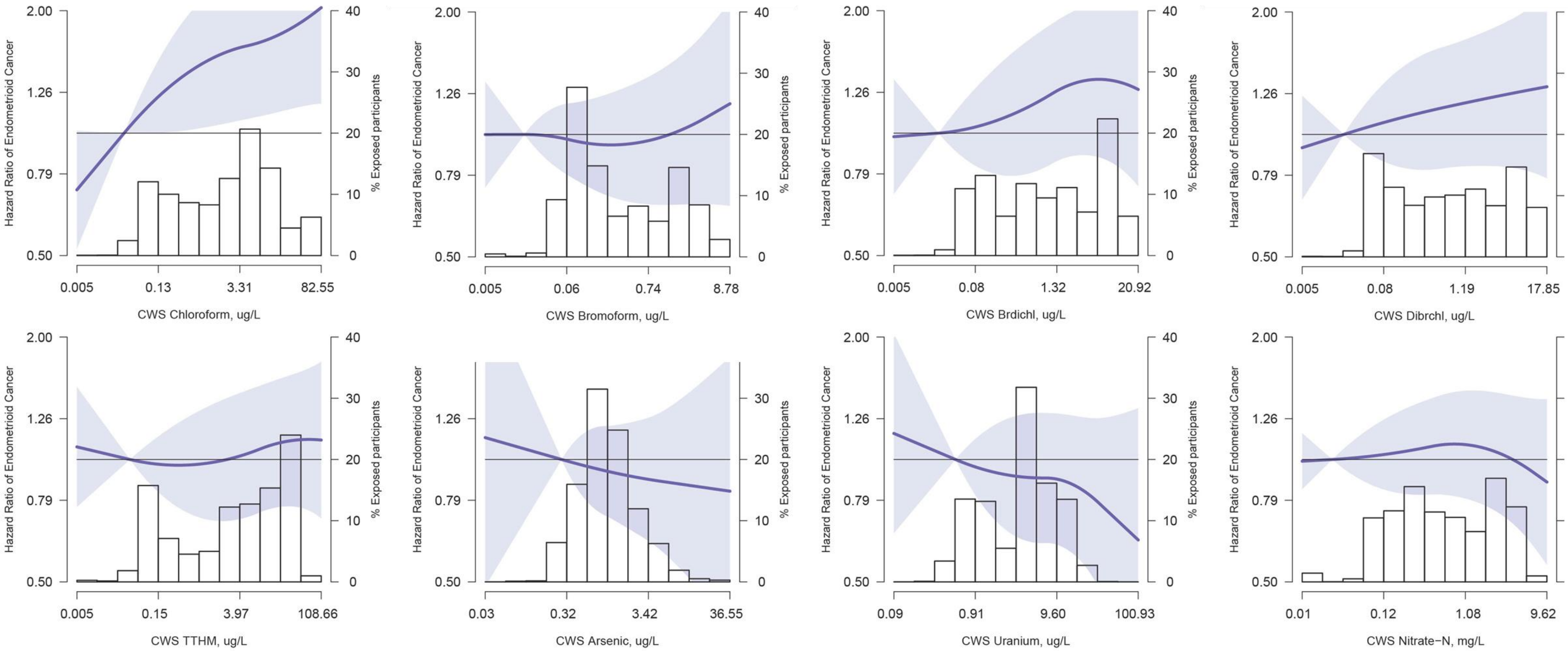


Lines with shaded areas represent the hazard ratios (HR) and 95% confidence intervals, based on cubic splines for Cox proportional hazards models using log2-transformed concentrations with knots at the 10$^{th}$ (reference), 50$^{th}$, and 90$^{th}$ percentiles. A black vertical line is at HR=1. The histogram represents the frequency distribution of exposures in the study sample. CWS exposures were linked to the address at enrollment. Analyses were restricted to participants with a residential duration at enrollment ≥ 10 years. Model was adjusted for baseline age (years) and baseline age$^2$, BMI category (<25 kg/m$^2$, 25-<30 kg/m$^2$, >30 kg/m$^2$, or missing), and smoking status (never, former, current, or missing).

© 2026 Spaur M et al. JAMA Network Open.

**eReferences**

1. Lubin JH, Colt, J. S., Camann, D., Davis, S., Cerhan, J. R., Severson, R. K., ... & Hartge, P. Epidemiologic evaluation of measurement data in the presence of detection limits. *Environmental health perspectives*. 2004;112(17):1691-1696.

2. Spaur M, Hurwitz, L. M., Medgyesi, D. N., Keil, A. P., Beane Freeman, L. E., ... & Ward, M. H. Exposures to drinking water contaminants in community water systems and risk of ovarian cancer in the California Teachers Study cohort. *Environmental Health Perspectives*. 2025;doi:DOI 10.1289/EHP16582

© 2026 Spaur M et al. JAMA Network Open.
