# Supplementary material for: Drinking Water Contaminant Exposures and Risk of Uterine Cancer
Source: JAMA Netw Open. 2026 Jul 22;9(7):e2624391. doi: 10.1001/jamanetworkopen.2026.24391 (PMC13392805; doi:10.1001/jamanetworkopen.2026.24391)
Supplement: Supplement 2. — Data Sharing Statement [file jamanetwopen-e2624391-s002.pdf]

Data Sharing Statement

Spaur. Drinking Water Contaminant Exposures and Risk of Uterine Cancer in the California Teachers Study Cohort. *JAMA Netw Open*. Published July 22, 2026.
doi:10.1001/jamanetworkopen.2026.24391

Data
Data available: No

Additional Information
Explanation for why data not available: All the data associated with this publication and in the California Teachers Study are available for research use. The California Teachers Study welcomes all such inquiries and encourages individuals to visit https://www.calteachersstudy.org/for-researchers.
